# Performance of the Global Diet Quality Score (GDQS) App in Predicting Nutrient Adequacy and Metabolic Risk Factors among Thai Adults

**DOI:** 10.1016/j.tjnut.2023.10.007

**Published:** 2023-10-14

**Authors:** Sabri Bromage, Tippawan Pongcharoen, Aree Prachansuwan, Pornpan Sukboon, Weerachat Srichan, Sasiumphai Purttiponthanee, Megan Deitchler, Mourad Moursi, Joanne Arsenault, Nazia Binte Ali, Carolina Batis, Wafaie W. Fawzi, Pattanee Winichagoon, Walter C. Willett, Wantanee Kriengsinyos

**Affiliations:** 1 Community Nutrition Unit, Institute of Nutrition, Mahidol University, Salaya, Phutthamonthon, Nakhon Pathom, Thailand; 2 Department of Global Health and Populations, Harvard T.H. Chan School of Public Health, Boston, MA, USA; 3 Department of Nutrition, Harvard T.H. Chan School of Public Health, Boston, MA, USA; 4 Human Nutrition Unit, Institute of Nutrition, Mahidol University, Salaya, Phutthamonthon, Nakhon Pathom, Thailand; 5 Research and Innovation Service Unit, Institute of Nutrition, Mahidol University, Salaya, Phutthamonthon, Nakhon Pathom, Thailand; 6 Intake Center for Dietary Assessment, Washington, DC, USA; 7 Health and Nutrition Research Center, National Institute of Public Health, Cuernavaca, Morelos, México; 8 Department of Epidemiology, Harvard T.H. Chan School of Public Health, Boston, MA, USA

**Keywords:** adults, diet quality metrics, dietary assessment, dietary diversity, GDQS, metabolic syndrome, nutrition surveillance, nutritional epidemiology, noncommunicable disease, nutrient adequacy

## Abstract

**Background:**

The Global Diet Quality Score (GDQS) was developed for monitoring nutrient adequacy and diet-related noncommunicable disease risk in diverse populations. A software application (GDQS app) was recently developed for the standardized collection of GDQS data. The application involves a simplified 24-h dietary recall (24HR) where foods are matched to GDQS-food groups using an onboard database, portion sizes are estimated at the food group level using cubic models, and the GDQS is computed.

**Objectives:**

The study aimed to estimate associations between GDQS scores collected using the GDQS app and nutrient adequacy and metabolic risks.

**Methods:**

In this cross-sectional study of 600 Thai males and nonpregnant/nonlactating females (40–60 y), we collected 2 d of GDQS app and paper-based 24HR, food-frequency questionnaires (FFQs), anthropometry, body composition, blood pressure, and biomarkers. Associations between application scores and outcomes were estimated using multiple regression, and application performance was compared with that of metrics scored using 24HR and FFQ data: GDQS, Minimum Dietary Diversity–Women, Alternative Healthy Eating Index–2010, and Global Dietary Recommendations score.

**Results:**

In covariate-adjusted models, application scores were significantly (*P* < 0.05) associated with higher energy-adjusted mean micronutrient adequacy computed using 24HR (range in estimated mean adequacy between score quintiles 1 and 5: 36.3%–44.5%) and FFQ (Q1–Q5: 40.6%–44.2%), and probability of protein adequacy from 24HR (Q1–Q5: 63%–72.5%). Application scores were inversely associated with BMI kg/m^2^ (Q1–Q5: 26.3–24.9), body fat percentage (Q1–Q5: 31.7%–29.1%), diastolic blood pressure (Q1–Q5: 84–81 mm Hg), and a locally-developed sodium intake score (Q1–Q5: 27.5–24.0 points out of 100); positively associated with high-density lipoprotein cholesterol (Q1–Q5: 49–53 mg/dL) and 24-h urinary potassium (Q1–Q5: 1385–1646 mg); and inversely associated with high midupper arm circumference (Q5/Q1 odds ratio: 0.52) and abdominal obesity (Q5/Q1 odds ratio: 0.51). Significant associations for the application outnumbered those for metrics computed using 24HR or FFQ.

**Conclusions:**

The GDQS app effectively assesses nutrient adequacy and metabolic risk in population surveys.

## Introduction

Assessment of diet and its contributions to health in populations is key to developing evidence-based strategies for improving diet quality and disease burden [1,2]. Diet surveys often employ 24-h diet recalls (24HRs) or food-frequency questionnaires (FFQs), which allow derivation of food and nutrient intakes. Data from 24HRs and FFQs can also be used to score metrics that summarize the joint contribution of multiple food groups and/or nutrients to health [3]; given the multifactorial drivers of malnutrition, such metrics provide valuable and easily conveyed complementary information for many applications. The Global Diet Quality Score (GDQS) was recently developed as a simple food-based metric for summarizing diet quality in diverse populations [4]. In secondary analysis of dietary data from 95,867 females of reproductive age in China, India, Mexico, the United States, and 10 African countries, the GDQS was associated with diverse outcomes related to nutrient adequacy and metabolic risk, and since development, the GDQS has been applied in secondary analysis of numerous surveys [[5], [6], [7], [8], [9], [10], [11], [12], [13], [14], [15], [16], [17], [18], [19], [20], [21]].

Despite the importance of food and nutrient intake data for decision-making, conventional dietary assessment methods require significant investments in adaptation to local food cultures, enumerator training, and data analysis, which may be costly and impractical in low- and middle-income countries (LMICs) [2,22], and data obtained from methods developed for different settings may not be immediately comparable. Thus, there is a need for innovative alternative methods that can generate robust and comparable data on population diet quality in LMICs.

A software application (GDQS app) was recently developed and designed by the Intake Center for Dietary Assessment in collaboration with Digital Development at FHI 360 to be an easy-to-use and economical tool that is purpose-built for collecting standardized GDQS data in population surveys [23]. The application is analyzed on a smartphone or tablet and consists of a user-friendly interface that guides an interviewer to administer a simplified 24HR. Foods that are recalled are automatically matched to corresponding GDQS-food groups using an extensive and customizable onboard food database assembled from international sources. After the recall, the list of consumed foods within each GDQS group is displayed back to the enumerator, who asks the participant to estimate the total consumed amount of all foods and beverages listed for each food group using a set of 10 cubes of various predefined sizes, the cubes are used to derive amounts of each GDQS-food group consumed in grams, and GDQS scores are computed using a validated algorithm [4,23]. The application is designed to be used in any country or context: it is free to use, is currently available in 9 languages, contains >7000 food items, and may be adapted to new populations and languages by translating the interface and food database and adding any local foods that may be missing (this process requires 2–3 mo) [24]. The application is particularly suited for use in limited-resource and LMIC settings: interviewers do not require nutrition training, an internet connection is not required to collect data, and the accompanying cubes are lightweight, stackable, and hence highly portable.

In this cross-sectional study, we characterized the performance of the GDQS app by estimating associations between the GDQS metric scored using the application and diverse outcomes reflective of nutrient adequacy and metabolic risk in a sample of 600 Thai adults. We also compared GDQS app performance with that of the following 4 existing metrics scored using 24HR and FFQ data collected from the same participants to understand the comparative utility of primary compared with secondary analysis of GDQS data and to provide a benchmark for the application’s performance: the GDQS itself, the Minimum Dietary Diversity–Women indicator (MDDW, a proxy for dietary micronutrient adequacy) [25], the Alternative Healthy Eating Index – 2010 (AHEI-2010, a proxy for diet-related chronic disease risk) [26], and the Global Dietary Recommendations score [GDR, which measures adherence to WHO dietary recommendations for preventing risk of noncommunicable diseases (NCDs)] [27].

## Methods

### Study population and sample size calculation

Participants were opportunistically sampled from the faculty and staff of the Salaya Campus of Mahidol University and the nearby general population (predominantly in Nakhon Pathom province and adjacent communities within the Bangkok metropolitan area) through advertisements posted at the university and 3 nearby health promotion centers. Eligible participants were literate, ethnically Thai males and nonpregnant, nonlactating females 40–60 y old. This age range was selected to avoid complexities introduced by pregnancy and lactation in females and frailty and decreased lean mass in both males and females, increase statistical power by allowing the inclusion of participants with a higher prevalence of NCD risk factors (which is important given the inherent challenge in examining cross-sectional associations between diet and NCDs, because of the latency period between dietary exposures and such outcomes), and given that numerous studies have found older participants and those with outcomes such as diabetes, dyslipidemia, and hypertension respond more positively to higher diet quality than younger and healthier populations [[28], [29], [30]]. Eligible participants were informed of the details of the study, and written informed consent was obtained prior to conducting assessments. The procedures followed were in accordance with the ethical standards of Mahidol University Central Institutional Review Board (IRB) (MU-CIRB 2021/404.1309) and Harvard Longwood Campus IRB (IRB21-0959).

Sample size calculations were informed by prior secondary analyses of 24HR data evaluating associations between the GDQS and metabolic syndrome (MetS) in 15–49-y-old urban females participating in the 2010–2012 China National Nutrition and Health Survey [31]. Based on available parameters, we estimated that a sample size of 555 participants (conservatively rounded to 600) would provide 80% power to conduct a 1-sided Cochrane-Armitage test for trend in multivariable odds of MetS across GDQS quintiles [32]. Given 600 participants and a 4.0 SD in the GDQS observed in prior analyses of the China National Nutrition and Health Survey [31], we estimated that we would also be powered to detect true correlation coefficients between the GDQS and continuous outcomes greater or equal to 0.13 [33].

### Dietary assessment

The GDQS app training materials, user interface, interview scripts, and food database were translated into Thai. Although the database includes foods from the ASEAN (Association of Southeast Asian Nations) Food Composition Database [34], we also reviewed Thai food composition tables [35,36] to identify and add local foods that might be missing. The application was rigorously pilot-tested among the research team prior to data collection.

On each of 2 visits spaced 1–2 wk apart, trained research assistants administered both the GDQS app and a paper-based multiple-pass 24HR to participants in a random order. Recall days included a weekday and weekend day for all participants. Portion size assessment in the 24HR utilized a measuring cup, household utensils, and a gram scale for weighing rice and sticky rice. A locally validated semiquantitative FFQ was also administered on the second visit to capture habitual diet over the past month [37]. Dual collection of 24HR and FFQ data allowed us to examine differences in metric scores derived from different assessment modalities and reference periods and estimate associations with nutrient intakes derived from the 2 methods (i.e., 24HR-derived metrics compared with 24HR nutrients, 24HR-derived metrics compared with FFQ nutrients, FFQ-derived metrics compared with 24HR nutrients, and FFQ-derived metrics compared with FFQ nutrients).

### Other assessments

A sociodemographic questionnaire, Global Physical Activity Questionnaire, Pittsburgh Sleep Quality Index (PSQI), tobacco-use questionnaire [[38], [39], [40]], and locally-developed sodium intake screener for Thai adults (assessing consumption frequency of 12 salty food groups and 10 questions on habits related to high salt intake over the past month) were collected at the first visit.

Height, midpoint waist circumference (WC), and midupper arm circumference (MUAC) were measured by trained nutritionists using a Harpenden stadiometer (Holtain, Ltd.) and Luftkin anthropometric tape (Apex Tool Group, Ltd.) according to standard protocols. Weight and percentage of body fat were measured via bio-electrical impedance using the InBody 270 device (InBody Co., Ltd.). Systolic and diastolic blood pressure (BP) were measured by sphygmomanometer according to WHO protocol. Venous blood was collected by registered nurses. Participants were instructed on how to collect their 24-h urine samples. A private laboratory was used to determine complete blood count and hemoglobin (Hb) via flow cytometry; fasting plasma glucose (FPG) via enzymatic method; HbA1C via turbidimetric inhibition immunoassay; triglycerides (TGs) and total, LDL, and HDL cholesterol via enzymatic colorimetric methods; and 24-h urinary sodium and potassium via indirect ion-selective electrode analysis. All anthropometric, BP, and biochemical assessments (except for 24-h urine) were collected on the first visit to balance the time commitment of participants.

The sequence of assessments is summarized in Supplemental Figure 1.

### Data processing

GDQS scores were computed using data from the FFQ (to produce the “GDQS-FFQ”), 24HR (to produce the “GDQS-24”), and GDQS app (to produce “app scores”). The FFQ and 24HR were also used to compute the MDDW (to produce the “MDDW-FFQ” and “MDDW-24”), and AHEI-2010 (to produce the “AHEI-FFQ” and “AHEI-24”), and the 24HR was also used to compute the GDR (to produce the “GDR-24”). The components and scoring method of the GDQS, MDDW, and AHEI-2010 are provided in published references [4,25,26] and compared in Table 2 of Bromage et al. 4], and the GDR is described in Herforth [27].

MDDW food groups were scored as “consumed” if total food group consumption met or exceeded 15 g [25]. Given inherent challenges in accurately measuring dietary sodium intake, the sodium intake screener was used to generate an overall sodium intake score (possible range: 0–100; range observed in this study: 2.9–53.8), which was used as an analytic outcome as well as a proxy for the AHEI-2010 sodium component in this analysis. The GDR was scored using only the foods present in the Thai diet quality questionnaire (DQQ) [41] according to the sentinel food approach described by Herforth et al., [42] and was not scored using FFQ data because the GDR’s dichotomous (yes/no) approach for scoring food groups would consider almost all food groups consumed to have been consumed over the FFQ reference period (1 mo). In this analysis, metrics were scored using only the first day of the GDQS app and 24HR to provide results representative of 24HR surveys in which 1 d of recall is collected from most or all participants.

Income was classified as above or below the 2021 national poverty line of 2803 Thai baht per month [43]. The metabolic equivalent of task minutes per week (MET-min/wk) and sleep quality were computed using the Global Physical Activity Questionnaire and PSQI criteria, respectively [38,39]. BMI was calculated and classified as underweight (<18.5), healthy (18.5–25), overweight (>25–30), or obese (>30) according to WHO criteria [44]. We defined abdominal adiposity as WC ≥90 cm for males and ≥80 cm for females [45] and separately as waist-to-height ratio >0.5 [46]; high MUAC as ≥30.9 cm for males and ≥30.0 cm for females [47]; hypertension as systolic BP ≥ 130 mm Hg, diastolic BP ≥ 85 mm Hg, or current use of hypertensive medications [45]; anemia as Hb <130 g/L for males and <120 g/L for females [48]; raised TG as >150 mg/dL [45], reduced HDL cholesterol as <40 mg/dL in males and <50 mg/dL in females [45], and raised LDL cholesterol as >160 mg/dL [49]; raised FPG as ≥100 mg/dL [45] and raised HbA1C as >6.5% [50]; and MetS according to International Diabetes Federation criteria, which consider 5 components: abdominal obesity, raised TG, reduced HDL cholesterol, hypertension, and raised FPG [45]. The 24-h urine samples were considered complete according to established criteria [51].

Nutrient intakes were calculated from the 24HR and FFQ using Thai and ASEAN food composition data [35,36]. Nutrient intakes from the 2 d of 24HRs were adjusted for within-person variation using the National Cancer Institute (NCI) method [52]. The full-probability method was applied to 24HR and FFQ nutrient intakes to estimate the probability of adequacy of 7 nutrients [53], based on estimated average requirements for the Thai population [54] (except for calcium, the estimated average requirement for which was obtained from proposed harmonized nutrient reference values [55]) and coefficients of variation in intake requirements from the Thai Dietary Reference Intakes (for vitamin A, iron, and zinc), United States National Academy of Medicine (for protein, thiamine, and vitamin B12) [56], and European Food Safety Administration (for calcium) [57]. The probability of adequacy of 6 micronutrients (vitamin A, thiamine, vitamin B12, calcium, iron, and zinc) was used to define the mean probability of micronutrient adequacy in 24HR and FFQ data. The 24HR and FFQ nutrient intakes and measures of nutrient adequacy were adjusted for total energy intake using the residual method [58].

### Statistical analysis

We compared mean metric scores (GDQS, MDDW, AHEI-2010, and GDR) derived from different data collection methods (GDQS app, 24HR, and FFQ) using analysis of variance, computed Spearman’s correlation between all metrics computed using all data collection tools, and concordance correlation between the same diet metrics computed using different tools [59]. Spearman’s correlations were estimated between diet metrics derived from different data collection methods and energy-adjusted nutrient intakes derived from 24HR and FFQ data, energy-adjusted mean probability of protein adequacy and energy-adjusted mean probability of micronutrient adequacy derived from 24HR and FFQ data, and continuous anthropometric, body composition, BP, and biomarker measurements. We estimated multivariable associations between metrics and outcomes using regression models adjusted for age, sex, education, MET-min/wk, smoking, PSQI score, and study group (Mahidol staff compared with community sample) to produce estimated marginal means (for continuous outcomes) or odds ratios (ORs) (for binary outcomes) for each metric quintile and *P* values for linear trend across quintiles. Metric-outcome associations were also estimated using models in which metrics were treated as continuous variables, and results were expressed in terms of a 1 SD difference in metrics.

To understand the comparative utility of GDQS app data compared with secondary analysis of the GDQS and other existing metrics scored using 24HR and FFQ data, we statistically compared the strengths of correlations and linear trends estimated between the application and outcomes with those estimated for the GDQS-24, GDQS-FFQ, and the MDDW, AHEI-2010, and GDR scored using 24HR and FFQ data. We also compared the performance of the GDQS-24 with that of other metrics scored using 24HR data and the GDQS-FFQ with that of other metrics scored using FFQ data to provide a benchmark for the performance of the GDQS metric against other metrics scored using the same instrument. Statistical comparisons were conducted using Wolfe’s tests for dependent correlation coefficients [60] and Wald tests of differences in parameter estimates between pairwise combinations of metrics following methods described in prior analyses [4,26].

## Results

### Sample characteristics

#### Demographic and nutritional profile

Eight hundred thirty-eight eligible participants were identified and invited to join the study, among which 600 provided consent and were enrolled. Data were largely complete for all measurements except for 24-h sodium and potassium, which were available for 408 participants only (Supplemental Table 1). Demographic and lifestyle characteristics and estimated usual nutrient intakes and adequacy computed from the 24HR of the study population are shown in Table 1, and clinical and biochemical characteristics are shown in Table 2. The mean age of participants was 49.3 + 5.7 y, 75.6% of participants were females, 24.8% of participants were employees of Mahidol University, and the rest were sampled from the nearby general population. MetS affected 42.5% of males and 35.9% of females according to International Diabetes Federation criteria, and 8.9% of males and 23.8% of females were anemic. A low to moderate prevalence of dietary adequacy of calcium (<0.2%), zinc (1.0%), vitamin B12 (19.1%), and vitamin A (63.9%) was observed in males and females, as was a moderate prevalence of iron adequacy in females (67.0%).

**Table 1:** Demographic and lifestyle characteristics and usual nutrient intakes and prevalence of nutrient adequacy of the study population by sex [mean (SD) or % (*n*)]

Characteristic	Total *n* = 600	Males *n* = 146		Females *n* = 454	
Age, y	49.3 (5.7)	49.6 (5.5)		49.2 (5.8)	
Mahidol staff, % (*n*)	24.8 (149)	25.3 (37)		18.7 (112)	
Education level, % (*n*)					
Primary school or below	19.3 (116)	17.1 (25)		20.0 (91)	
Secondary school or high school	24.8 (149)	28.1 (41)		23.8 (108)	
Professional diploma or university	55.8 (335)	54.8 (80)		56.2 (255)	
Married, % (*n*)	61.5 (369)	70.5 (103)		58.6 (266)	
Buddhist, % (*n*)	97.3 (584)	95.2 (139)		98.0 (445)	
Employed, % (*n*)	88.3 (530)	93.2 (136)		86.8 (394)	
Mean household size	3.6 (1.7)	3.7 (1.8)		3.5 (1.6)	
Annual household income, 1000 Thai baht^1^	396 (200, 720)	480 (230, 800)		370 (200, 684)	
Per capita household income/mo, 1000 Thai baht	10 (5, 20)	11 (5, 21)		10 (5, 20)	
Individual income/mo, 1000 Thai baht	17 (10, 32)	20 (12, 37)		17 (9, 30)	
Individual income below the national poverty line, % (*n*)	2.0 (11)	1.5 (2)		2.2 (9)	
Physical activity, MET-min/wk	460 (120, 1190)	660 (240, 1680)		420 (120, 1105)	
Sedentary time, min/d	240 (120, 360)	240 (120, 360)		210 (120, 360)	
Current smoker, % (*n*)	6.2 (37)	20.5 (30)		1.5 (7)	
Poor sleep quality (PSQI score >5), % (*n*)	53.5 (321)	51.4 (75)		45.8 (246)	
Sodium screener score (range: 0–100)	24.4 (9.3)	26.0 (9.2)		23.9 (9.2)	
Underlying conditions, % (*n*)					
Diabetes mellitus	7.5 (45)	8.2 (12)		7.3 (33)	
Hypertension	19.0 (114)	22.6 (33)		17.8 (81)	
Dyslipidemia	19.8 (119)	20.5 (30)		19.6 (89)	
Nutrient intakes		**Mean (SD)**	**% Adequacy**	**Mean (SD)**	**% Adequacy**
Energy, kcal/d		1871 (412)		1463 (353)	
Carbohydrate, g/d		262.8 (62.8)		204.3 (52.2)	
Total sugar, g/d		64.1 (18.1)		58.8 (15.8)	
Dietary fiber, g/d		10.9 (2.5)		10.1 (2.4)	
Protein, g/d		72.7 (16.5)	76.58	59.3 (15.6)	70.02
Animal-source protein, % of total protein		66.6 (6.8)		66.1 (9.3)	
Fat, g/d		55.9 (12.9)		46.8 (12.2)	
Contributors to total energy intake					
Carbohydrate, %		56.3 (5.9)		56.1 (6.8)	
Protein, %		15.7 (2.4)		16.4 (3.2)	
Fat, %		27.2 (4.1)		29.0 (4.4)	
Saturated fat, %		10.1 (1.7)		11.1 (1.8)	
Unsaturated fat, %		17.1 (3.0)		17.9 (3.1)	
Calcium, mg/d		346.6 (69.0)	0.02	329.3 (79.5)	0.22
Iron, mg/d		10.5 (1.7)	99.36	9.3 (1.7)	66.97
Animal-source iron, % of total iron		41.9 (5.5)		41.1 (6.7)	
Sodium, mg/d		2527.0 (438.0)		2257.3 (385.8)	
Zinc, mg/d		5.1 (1.2)	1.33	4.2 (1.0)	0.91
Vitamin A, RAE/d		511.8 (104.1)	51.04	493.7 (103.4)	68.10
Thiamine, mg/d		1.3 (0.2)	89.62	1.2 (0.2)	87.59
Vitamin B12, *μ*g/d		1.6 (0.6)	24.43	1.5 (0.6)	17.47

Population characteristics are presented as mean (SD) or % (*n*) except for^1^ which is presented as median (P25, P75). Nutrient intake statistics are estimated from 2-d of 24-h dietary recalls per person adjusted for within-person variation. Percentage adequacy is estimated using the full-probability method.

Abbreviations: MET, metabolic equivalent of task; P25, P75; 25th and 75th percentiles; PSQI, Pittsburgh sleep quality index; RAE, retinol activity equivalent; SD standard deviation.

**Table 2:** Clinical and biochemical characteristics of the study population [mean (SD) or % (*n*)]

Characteristic	Total (*n* = 600)	Males (*n* = 146)	Females (*n* = 454)
Weight, kg	64.8 (13.6)	73.7 (13.0)	61.9 (12.6)
Height, cm	159.1 (7.8)	168.8 (5.6)	156.0 (5.6)
BMI, kg/m^2^	25.5 (4.7)	25.9 (4.2)	25.4 (4.9)
Underweight (<18.5), % (*n*)	1.7 (10)	0.7 (1)	2.0 (9)
Normal (18 to <25), % (*n*)	50.7 (304)	50.0 (73)	50.9 (231)
Overweight (25 to <30), % (*n*)	32.2 (194)	33.6 (49)	31.9 (145)
Obese (≥30), % (*n*)	15.3 (92)	15.8 (23)	15.2 (69)
MUAC, cm	30.4 (4.1)	31.5 (3.5)	30.0 (4.2)
High MUAC (≥30.9 cm in males, ≥30.0 cm in females), % (*n*)	47.1 (282)	55.5 (81)	44.4 (201)
WC, cm	85.0 (12.2)	90.1 (11.7)	83.3 (11.9)
Abdominal obesity (WC ≥90 cm in males, ≥80 cm in females), % (*n*)	52.7 (316)	48.6 (71)	54.0 (245)
Waist-to-height ratio >0.5, % (*n*)	62.8 (377)	66.4 (97)	61.7 (280)
Fat mass, %	33.1 (8.1)	25.5 (6.5)	35.6 (7.0)
SBP, mm Hg	121 (18)	128 (15)	119 (18)
DBP, mm Hg	80 (12)	84 (11)	79 (12)
Hypertension (BP ≥130/85 mm Hg or on medication), % (*n*)	43.0 (258)	54.1 (79)	39.4 (179)
Hb, g/L	130.1 (15.3)	144.9 (12.1)	125.4 (13.0)
Anemia (Hb <130 g/L in males, <120 g/L in females), % (*n*)	20.2 (121)	8.9 (13)	23.8 (108)
Total cholesterol, mg/dL	213 (43)	209 (43)	214 (43)
LDL cholesterol, mg/dL	147 (41)	141 (39)	148 (42)
Raised LDL cholesterol (>160 mg/dL), % (*n*)	34.3 (206)	32.9 (48)	34.8 (158)
HDL cholesterol, mg/dL	53 (12)	47 (11)	55 (12)
Reduced HDL cholesterol (<40 mg/dL in males, <50 mg/dL in females), % (*n*)	31.8 (191)	21.9 (32)	35.0 (159)
TG, mg/dL^1^	106 (74, 158)	126 (87, 179)	102 (71, 152)
Raised TG (>150 mg/dL), % (*n*)	40.2 (241)	52.1 (76)	36.3 (165)
FPG, mg/dL	105 (34)	108 (29)	105 (35)
Raised FPG (≥ 100 mg/dL), % (*n*)	39.8 (239)	51.4 (75)	36.1 (164)
HbA1C, %	5.8 (1.4)	5.8 (1.1)	5.9 (1.5)
HbA1C >6.5%, *n* (%)	11.0 (66)	12.3 (18)	10.6 (48)
Metabolic syndrome (IDF criteria), % (*n*)	37.5 (225)	42.5 (62)	35.9 (163)
24-h urinary sodium, mg	3397 (1424)	3705 (1635)	3282 (1320)
24-h urinary potassium, mg	1525 (562)	1564 (608)	1513 (542)

Values are presented as mean (SD) or % (*n*) except for ^1^ which is presented as median (P25, P75).

Abbreviations: BMI, body mass index; BP, blood pressure; DBP, diastolic blood pressure; FPG, fasting plasma glucose; Hb, hemoglobin; HbA1C, hemoglobin A1C; HDL, high-density lipoprotein; IDF, International Diabetes Federation; LDL, low-density lipoprotein; MUAC, midupper arm circumference; P25, P75; 25th and 75th percentiles; SBP, systolic blood pressure; SD, standard deviation; TG, triglyceride; WC, waist circumference.

#### Metric scores

Mean GDQS-FFQ scores exceeded mean GDQS app scores in females, app scores exceeded 24HR scores in males and females, and scores of all FFQ-derived metrics exceeded those derived from the 24HR in males and females (*P* < 0.05) (Supplemental Table 2). Higher GDQS-24, GDQS-FFQ, GDR-24, and AHEI-FFQ scores were found in females than males (*P* < 0.05). Supplemental Table 3 provides a Spearman’s correlation matrix of metric scores, and Supplemental Table 4 provides a concordance correlation between the same metrics computed using different data collection tools. Concordance was highest between the GDQS app GDQS-24 (*r* = 0.49) and weaker between the GDQS app and GDQS-FFQ (*r* = 0.22) and the GDQS-24 and GDQS-FFQ (0.15). Different metrics computed from the same instrument (24HR or FFQ) generally correlated better with one another than with the same metric computed using a different instrument (for example, the pairwise correlation between the GDQS-FFQ, MDDW-FFQ, and AHEI-FFQ was 0.75, whereas the correlation between each of these metrics and their 24HR counterparts was 0.21, 0.18, and 0.50, respectively).

### Comparing the performance of the GDQS app, GDQS-24, and GDQS-FFQ in Spearman’s correlations with energy-adjusted nutrient intakes and adequacy

#### Correlations between GDQS metrics compared with 24HR nutrients

In analysis of energy-adjusted nutrient intakes and adequacy computed from 24HR data, GDQS app scores were positively correlated with intakes of protein (*r* = 0.09), polyunsaturated fat (0.12), fiber (0.32), calcium (0.22), iron (0.22), zinc (0.14), vitamin A (0.26), thiamine (0.10), and vitamin B12 (0.16); probability of protein adequacy (0.10) and mean probability of micronutrient adequacy (0.26); and negatively correlated with intake of saturated fat (–0.15) (*P* < 0.05) (Table 3). Performance of the GDQS-24 and GDQS-FFQ in predicting energy-adjusted 24HR nutrient intakes and adequacy was less consistent: the GDQS-24 was uncorrelated with protein; the GDQS-FFQ was uncorrelated with zinc, thiamine, and vitamin B12; and neither metric was correlated with saturated fat or the probability of protein adequacy (*P ≥* 0.05). Correlations were stronger for the GDQS app than the GDQS-24 for saturated fat and stronger for the GDQS app than the GDQS-FFQ for saturated fat and zinc (*P* for difference between metrics <0.05).

**Table 3:** Statistical comparison of Spearman’s correlation coefficients between methods for collecting GDQS data and outcomes related to nutrient adequacy and metabolic risk among Thai adults: Global Diet Quality Score (GDQS) app compared with GDQS-24 compared with GDQS-food-frequency questionnaire

Outcome	r_s_ (*P*)			*P*-diff		
	GDQS app	GDQS-24	GDQS-FFQ	GDQS app vs. GDQS-24	GDQS app vs. GDQS-FFQ	GDQS-24 vs. GDQS-FFQ
Energy-adjusted 24HR nutrient intakes and adequacy						
Protein	0.09 (0.032)∗	0.07 (0.097)	0.12 (0.004)∗	0.549	0.530	0.316
Saturated fat	–0.15 (<0.001)∗	0.01 (0.807)	0.03 (0.479)	<0.001∗	<0.001∗	0.794
Monounsaturated fat	–0.01 (0.866)	0.07 (0.097)	0.03 (0.462)	0.058	0.321	0.451
Polyunsaturated fat	0.12 (0.002)∗	0.11 (0.005)∗	0.10 (0.025)∗	0.678	0.706	0.682
Fiber	0.32 (<0.001)∗	0.34 (<0.001)∗	0.26 (<0.001)∗	0.986	0.242	0.071
Calcium	0.22 (<0.001)∗	0.19 (<0.001)∗	0.21 (<0.001)∗	0.500	0.847	0.713
Iron	0.22 (<0.001)∗	0.16 (<0.001)∗	0.14 (0.001)∗	0.090	0.113	0.767
Zinc	0.14 (<0.001)∗	0.14 (0.001)∗	0.02 (0.627)	0.867	0.015∗	0.023∗
Vitamin A	0.26 (<0.001)∗	0.20 (<0.001)∗	0.21 (<0.001)∗	0.180	0.353	0.744
Thiamine	0.10 (0.016)∗	0.15 (<0.001)∗	0.07 (0.077)	0.343	0.919	0.118
Vitamin B12	0.16 (<0.001)∗	0.13 (0.002)∗	0.07 (0.089)	0.378	0.077	0.298
Probability of protein adequacy	0.10 (0.012)∗	0.07 (0.081)	0.07 (0.087)	0.314	0.491	0.981
Mean probability of micronutrient adequacy	0.26 (<0.001)∗	0.23 (<0.001)∗	0.18 (<0.001)∗	0.464	0.205	0.412
Energy-adjusted FFQ nutrient intakes and adequacy						
Protein	–0.05 (0.245)	0.04 (0.355)	0.09 (0.036)∗	0.066	0.008∗	0.415
Saturated fat	–0.02 (0.698)	0.11 (0.010)∗	0.07 (0.085)	0.001∗	0.157	0.347
Monounsaturated fat	–0.02 (0.705)	0.09 (0.036)∗	0.04 (0.346)	0.016∗	0.266	0.336
Polyunsaturated fat	0.03 (0.494)	0.14 (0.001)∗	0.17 (<0.001)∗	0.013∗	0.004∗	0.500
Fiber	0.20 (<0.001)∗	0.18 (<0.001)∗	0.49 (<0.001)∗	0.588	<0.001∗	<0.001∗
Calcium	0.12 (0.004)∗	0.15 (<0.001)∗	0.49 (<0.001)∗	0.362	<0.001∗	<0.001∗
Iron	0.10 (0.020)∗	0.17 (<0.001)∗	0.38 (<0.001)∗	0.084	<0.001∗	<0.001∗
Zinc	0.01 (0.793)	0.10 (0.015)∗	0.17 (<0.001)∗	0.033∗	0.001∗	0.161
Vitamin A	0.11 (0.009)∗	0.14 (0.001)∗	0.11 (0.007)∗	0.673	0.935	0.442
Thiamine	0.01 (0.781)	0.12 (0.004)∗	0.26 (<0.001)∗	0.009∗	<0.001∗	0.009∗
Vitamin B12	0.04 (0.399)	0.09 (0.034)∗	0.01 (0.789)	0.279	0.693	0.173
Probability of protein adequacy	0.04 (0.372)	0.04 (0.336)	0.21 (<0.001)∗	0.898	<0.001∗	0.001∗
Mean probability of micronutrient adequacy	0.10 (0.012)∗	0.16 (<0.001)∗	0.46 (<0.001)∗	0.149	<0.001∗	<0.001∗
Clinical and biochemical measurements						
BMI	–0.11 (0.010)∗	–0.07 (0.073)	–0.05 (0.199)	0.455	0.242	0.480
Fat mass, %	–0.11 (0.007)∗	0.01 (0.798)	–0.04 (0.353)	0.003∗	0.115	0.591
MUAC	–0.11 (0.005)∗	–0.10 (0.016)∗	–0.07 (0.094)	0.774	0.285	0.399
WC	–0.10 (0.015)∗	–0.10 (0.013)∗	–0.09 (0.039)∗	0.972	0.676	0.607
SBP	–0.01 (0.755)	–0.05 (0.192)	–0.04 (0.280)	0.228	0.606	0.707
DBP	–0.08 (0.051)	–0.10 (0.017)∗	–0.08 (0.071)	0.517	0.791	0.393
Hb	0.01 (0.768)	–0.09 (0.029)∗	–0.04 (0.286)	0.013∗	0.298	0.557
Total cholesterol	–0.01 (0.779)	0.00 (0.984)	–0.04 (0.295)	0.977	0.790	0.392
LDL cholesterol	–0.05 (0.233)	–0.02 (0.623)	–0.03 (0.543)	0.735	0.387	0.864
HDL cholesterol	0.09 (0.034)∗	0.09 (0.023)∗	0.09 (0.031)∗	0.932	0.742	0.992
TG	–0.10 (0.017)∗	–0.08 (0.055)	–0.08 (0.060)	0.698	0.565	0.789
FPG	–0.07 (0.079)	–0.08 (0.060)	–0.02 (0.582)	0.978	0.313	0.301
HbA1C	0.00 (0.917)	0.02 (0.671)	0.00 (0.960)	0.678	0.953	0.692
Number of MetS components	–0.07 (0.087)	–0.05 (0.182)	–0.07 (0.077)	0.652	0.951	0.931
24-h urinary sodium	0.02 (0.713)	–0.07 (0.153)	–0.06 (0.256)	0.021∗	0.329	0.683
24-h urinary potassium	0.18 (<0.001)∗	0.12 (0.017)∗	0.21 (<0.001)∗	0.131	0.854	0.275
Sodium screener score	–0.19 (<0.001)∗	–0.15 (<0.001)∗	–0.08 (0.047)∗	0.361	0.030∗	0.117

*P*-diff: *P* for difference estimated using Wolfe’s test for dependent correlation coefficients. ∗ indicates *P* for the significance of correlation coefficient <0.05 or *P-*diff <0.05.

Abbreviations: BMI, body mass index; DBP, diastolic blood pressure; FFQ/-FFQ, food-frequency questionnaire; FPG, fasting plasma glucose; GDQS, Global Diet Quality Score; Hb, hemoglobin; HbA1C, hemoglobin A1C; HDL, high-density lipoprotein; LDL, low-density lipoprotein; MetS, metabolic syndrome; MUAC, midupper arm circumference; SBP, systolic blood pressure; TG, triglyceride; WC, waist circumference

24HR/-24, 24-h dietary recall.

#### Correlations between GDQS metrics compared with FFQ nutrients

In analysis of energy-adjusted nutrient intakes and adequacy computed from FFQ data, the GDQS app and GDQS-24 were correlated with intakes of fiber, calcium, iron, vitamin A, and mean probability of micronutrient adequacy (range: *r* = 0.10–0.20) whereas the GDQS-24 was further correlated with intakes of zinc, thiamine, vitamin B12, and all fatty acids (*r* = 0.09–0.14), including saturated fat in an undesired direction (*r* = 0.11) (*P* < 0.05) (Table 3). The GDQS-FFQ was positively correlated with intakes of all nutrients (*r* = 0.09–0.49; *P* < 0.05) except saturated fat, monounsaturated fat, and vitamin B12, and also correlated with probability of protein adequacy (*r* = 0.21) and mean micronutrient adequacy (0.46). Correlations were significantly stronger for the GDQS-24 than the GDQS app for intakes of all fatty acids (including saturated fat), zinc, and thiamine, and stronger for the GDQS-FFQ than the application for all nutrients except saturated and monounsaturated fat, vitamin A, and vitamin B12 (*P* < 0.05).

### Comparing the performance of the GDQS app, GDQS-24, and GDQS-FFQ with that of the MDDW, AHEI, and GDR scored using 24HR and FFQ data in Spearman’s correlations with energy-adjusted nutrient intakes and adequacy

#### Correlations between GDQS and non-GDQS metrics compared with 24HR nutrients

In analyses comparing the GDQS app with non-GDQS metrics scored using 24HR and FFQ data, the GDQS app was more strongly correlated than the MDDW-24 and MDDW-FFQ with energy-adjusted 24HR intakes of saturated fat, fiber, and zinc, and more strongly correlated than the MDDW-FFQ with energy-adjusted 24HR iron and vitamin B12 intakes and mean probability of micronutrient adequacy (*P* < 0.05) (Table 4). In comparison with the GDR-24, the GDQS app was more strongly correlated with energy-adjusted 24HR intakes of protein, polyunsaturated fat, iron, and probability of protein adequacy (*P* < 0.05). The GDQS app was more strongly correlated than the AHEI-24 and AHEI-FFQ with energy-adjusted 24HR intakes of saturated fat and vitamin B12, more strongly correlated than the AHEI-24 with iron intake, and more strongly correlated than the AHEI-FFQ with 24HR intake of zinc (*P* < 0.05). The AHEI-24 outperformed the GDQS app only in correlations with energy-adjusted 24HR intakes of polyunsaturated fat and fiber (*P* < 0.05). In comparing the GDQS-24 with non-GDQS metrics, no differences were observed in correlations with energy-adjusted probability of protein or mean micronutrient adequacy computed from 24HR data (*P* < 0.05) (Supplemental Table 5), whereas the GDQS-FFQ outperformed the MDDW-FFQ in correlations with energy-adjusted mean probability of micronutrient adequacy computed from the 24HR (*P* < 0.05).

**TABLE 4:** Statistical comparison of Spearman’s correlations between diet metrics and outcomes related to nutrient adequacy and metabolic risk among Thai adults: Global Diet Quality Score (GDQS) app compared with non-GDQS metrics scored using 24-h dietary recall and food-frequency questionnaire data

Outcome	r_s_ (*P*)						*P*-diff				
	GDQS app	MDDW-24	AHEI-24	GDR-24	MDDW-FFQ	AHEI-FFQ	GDQS app vs. MDDW-24	GDQS app vs. AHEI-24	GDQS app vs. GDR-24	GDQS app vs. MDDW-FFQ	GDQS app vs. AHEI-FFQ
Energy-adjusted 24HR nutrient intakes and adequacy											
Protein	0.09 (0.032)∗	0.07 (0.088)	0.04 (0.385)	–0.01 (0.752)	0.07 (0.098)	0.07 (0.105)	0.315	0.257	0.026∗	0.683	0.744
Saturated fat	–0.15 (<0.001)∗	0.04 (0.362)	–0.05 (0.248)	–0.14 (<0.001)∗	0.06 (0.166)	–0.05 (0.253)	0.000∗	0.040∗	0.543	0.000∗	0.038∗
Monounsaturated fat	–0.01 (0.866)	0.07 (0.090)	0.05 (0.219)	–0.07 (0.082)	0.05 (0.259)	–0.02 (0.685)	0.148	0.137	0.116	0.315	0.821
Polyunsaturated fat	0.12 (0.002)∗	0.10 (0.011)∗	0.24 (<0.001)∗	0.03 (0.436)	0.12 (0.004)∗	0.11 (0.010)∗	0.425	0.001∗	0.010∗	0.955	0.961
Fiber	0.32 (<0.001)∗	0.23 (<0.001)∗	0.46 (<0.001)∗	0.39 (<0.001)∗	0.18 (<0.001)∗	0.38 (<0.001)∗	0.015	0.001∗	0.248	0.006∗	0.218
Calcium	0.22 (<0.001)∗	0.21 (<0.001)∗	0.24 (<0.001)∗	0.18 (<0.001)∗	0.20 (<0.001)∗	0.25 (<0.001)∗	0.724	0.666	0.432	0.680	0.542
Iron	0.22 (<0.001)∗	0.16 (<0.001)∗	0.10 (0.010)∗	0.10 (0.011)∗	0.08 (0.063)	0.13 (0.001)∗	0.099	0.003∗	0.004∗	0.007∗	0.056
Zinc	0.14 (<0.001)∗	0.06 (0.130)	0.08 (0.052)	0.12 (0.003)∗	0.00 (0.867)	0.03 (0.500)	0.024∗	0.080	0.449	0.003∗	0.012∗
Vitamin A	0.26 (<0.001)∗	0.26 (<0.001)∗	0.19 (<0.001)∗	0.18 (<0.001)∗	0.16 (<0.001)∗	0.24 (<0.001)∗	0.788	0.065	0.071	0.061	0.498
Thiamine	0.10 (0.016)∗	0.16 (<0.001)∗	0.07 (0.081)	0.11 (0.008)∗	0.05 (0.191)	0.02 (0.547)	0.206	0.398	0.867	0.558	0.218
Vitamin B12	0.16 (<0.001)∗	0.16 (<0.001)∗	0.03 (0.442)	0.09 (0.021)∗	0.05 (0.223)	0.07 (0.104)	0.548	0.000∗	0.126	0.035∗	0.029∗
Probability of protein adequacy	0.10 (0.012)∗	0.06 (0.124)	0.06 (0.141)	0.01 (0.735)	0.09 (0.040)∗	0.10 (0.016)∗	0.233	0.319	0.034∗	0.695	0.951
Mean probability of micronutrient adequacy	0.26 (<0.001)∗	0.25 (<0.001)∗	0.21 (<0.001)∗	0.19 (<0.001)∗	0.10 (0.015)∗	0.20 (<0.001)∗	0.505	0.153	0.140	0.003∗	0.287
Energy-adjusted FFQ nutrient intakes and adequacy											
Protein	–0.05 (0.245)	0.00 (0.987)	0.03 (0.520)	0.02 (0.608)	0.02 (0.584)	0.05 (0.238)	0.221	0.058	0.067	0.080	0.049∗
Saturated fat	–0.02 (0.698)	0.04 (0.367)	0.01 (0.751)	–0.06 (0.156)	0.10 (0.016)∗	0.02 (0.692)	0.155	0.217	0.467	0.031∗	0.526
Monounsaturated fat	–0.02 (0.705)	0.04 (0.307)	0.01 (0.721)	0.00 (0.973)	0.01 (0.873)	0.01 (0.758)	0.130	0.358	0.569	0.394	0.534
Polyunsaturated fat	0.03 (0.494)	0.03 (0.453)	0.09 (0.024)∗	0.06 (0.165)	0.09 (0.026)∗	0.19 (<0.001)∗	0.876	0.122	0.448	0.093	0.001∗
Fiber	0.20 (<0.001)∗	0.12 (0.004)∗	0.34 (<0.001)∗	0.27 (<0.001)∗	0.33 (<0.001)∗	0.62 (<0.001)∗	0.039∗	0.001∗	0.072	0.010∗	0.000∗
Calcium	0.12 (0.004)∗	0.13 (0.002)∗	0.16 (<0.001)∗	0.19 (<0.001)∗	0.45 (<0.001)∗	0.45 (<0.001)∗	0.946	0.407	0.022∗	0.000∗	0.000∗
Iron	0.10 (0.020)∗	0.09 (0.029)∗	0.20 (<0.001)∗	0.02 (<0.001)∗	0.26 (<0.001)∗	0.37 (<0.001)∗	0.784	0.010∗	0.002∗	0.002∗	0.000∗
Zinc	0.01 (0.793)	0.08 (0.059)	0.08 (0.057)	0.07 (0.102)	0.11 (0.008)∗	0.12 (0.003)∗	0.183	0.132	0.214	0.035∗	0.045
Vitamin A	0.11 (0.009)∗	0.11 (0.007)∗	0.15 (<0.001)∗	0.20 (<0.001)∗	0.04 (0.356)	0.14 (<0.001)∗	0.868	0.227	0.011∗	0.203	0.267
Thiamine	0.01 (0.781)	0.08 (0.045)∗	0.15 (<0.001)∗	0.12 (0.003)∗	0.17 (<0.001)∗	0.27 (<0.001)∗	0.156	0.001∗	0.005∗	0.001∗	0.000∗
Vitamin B12	0.04 (0.399)	0.05 (0.238)	0.02 (0.703)	0.10 (0.020)∗	-0.01 (0.767)	-0.02 (0.627)	0.742	0.670	0.166	0.552	0.301
Probability of protein adequacy	0.04 (0.372)	0.08 (0.042)∗	0.01 (0.782)	0.03 (0.515)	0.18 (<0.001)∗	0.14 (<0.001)∗	0.282	0.500	0.888	0.001∗	0.032∗
Mean probability of micronutrient adequacy	0.10 (0.012)∗	0.17 (<0.001)∗	0.17 (<0.001)∗	0.19 (<0.001)∗	0.38 (<0.001)∗	0.39 (<0.001)∗	0.194	0.107	0.042∗	0.000∗	0.000∗
Clinical and biochemical measurements											
BMI	–0.11 (0.010)∗	–0.06 (0.176)	–0.08 (0.054)	–0.04 (0.369)	–0.08 (0.041)∗	–0.13 (0.002)∗	0.276	0.541	0.131	0.766	0.865
Fat mass, %	–0.11 (0.007)∗	–0.02 (0.620)	–0.04 (0.317)	0.00 (0.972)	–0.05 (0.222)	–0.06 (0.147)	0.057	0.150	0.009∗	0.231	0.116
MUAC	–0.11 (0.005)∗	–0.07 (0.090)	–0.07 (0.075)	–0.06 (0.137)	–0.09 (0.030)∗	–0.13 (0.001)∗	0.336	0.234	0.287	0.701	0.934
WC	–0.10 (0.015)∗	–0.05 (0.215)	–0.09 (0.033)∗	–0.06 (0.123)	–0.10 (0.012)∗	–0.17 (<0.001)∗	0.262	0.764	0.552	0.830	0.214
SBP	–0.01 (0.755)	–0.04 (0.301)	0.02 (0.660)	–0.02 (0.576)	–0.09 (0.024)∗	–0.07 (0.111)	0.592	0.322	0.658	0.109	0.394
DBP	–0.08 (0.051)	–0.06 (0.178)	–0.04 (0.277)	–0.08 (0.060)	–0.12 (0.004)∗	–0.11 (0.009)∗	0.464	0.342	0.792	0.468	0.819
Hb	0.01 (0.768)	–0.05 (0.250)	–0.03 (0.479)	–0.03 (0.476)	–0.06 (0.185)	–0.05 (0.200)	0.250	0.559	0.308	0.152	0.133
Total cholesterol	–0.01 (0.779)	–0.07 (0.106)	0.03 (0.517)	–0.07 (0.075)	–0.04 (0.371)	–0.01 (0.837)	0.275	0.318	0.227	0.642	0.744
LDL cholesterol	–0.05 (0.233)	–0.08 (0.061)	–0.02 (0.657)	–0.07 (0.083)	–0.02 (0.635)	–0.02 (0.577)	0.542	0.374	0.688	0.433	0.457
HDL cholesterol	0.09 (0.034)∗	0.08 (0.038)∗	0.12 (0.003)∗	0.06 (0.165)	0.08 (0.041)∗	0.16 (<0.001)∗	0.886	0.516	0.595	0.957	0.080
TG	–0.10 (0.017)∗	–0.07 (0.078)	–0.08 (0.041)∗	–0.09 (0.020)∗	–0.09 (0.038)∗	–0.16 (<0.001)∗	0.465	0.774	0.848	0.847	0.292
FPG	–0.07 (0.079)	–0.08 (0.048)∗	–0.07 (0.090)	–0.06 (0.134)	–0.02 (0.641)	–0.06 (0.158)	0.858	0.747	0.789	0.436	0.676
HbA1C	0.00 (0.917)	0.01 (0.825)	0.03 (0.529)	–0.02 (0.667)	0.01 (0.863)	–0.03 (0.419)	0.910	0.527	0.601	0.979	0.532
Number of MetS components	–0.07 (0.087)	–0.07 (0.100)	–0.04 (0.361)	0.00 (0.895)	–0.11 (0.007)∗	–0.12 (0.003)∗	0.811	0.320	0.082	0.343	0.340
24-h urinary sodium	0.02 (0.713)	0.02 (0.680)	–0.07 (0.166)	–0.08 (0.098)	–0.03 (0.512)	–0.14 (0.004)∗	0.831	0.073	0.033∗	0.489	0.007∗
24-h urinary potassium	0.18 (<0.001)∗	0.13 (0.009)∗	0.22 (<0.001)∗	0.13 (0.010)∗	0.13 (0.008)∗	0.26 (<0.001)∗	0.260	0.657	0.346	0.326	0.327
Sodium screener score	–0.19 (<0.001)∗	–0.11 (0.010)∗	–0.47 (<0.001)∗	–0.23 (<0.001)∗	0.02 (0.562)	–0.45 (<0.001)∗	0.039∗	0.000∗	0.302	0.000∗	0.000∗

*P*-diff: *P* for difference estimated using Wolfe’s test for dependent correlation coefficients. ∗ indicates *P* for the significance of correlation coefficient <0.05 or *P*-diff <0.05.

Abbreviations: AHEI, Alternative Healthy Eating Index; BMI, body mass index; DBP, diastolic blood pressure; FFQ / -FFQ, food-frequency questionnaire; FPG, fasting plasma glucose; GDQS, Global Diet Quality Score; GDR, Global Dietary Recommendations; Hb, hemoglobin; HbA1C, hemoglobin A1C; HDL, high-density lipoprotein; LDL, low-density lipoprotein; MDDW, Minimum Diet Diversity–Wwomen; MetS, metabolic syndrome, MUAC, midupper arm circumference; SBP, systolic blood pressure; TG, triglyceride; WC, waist circumference; 24HR / -24, 24-h dietary recall.

#### Correlations between GDQS and non-GDQS metrics compared with FFQ nutrients

In analysis of energy-adjusted nutrient intakes and adequacy computed from the FFQ, the MDDW-FFQ and AHEI-FFQ outperformed the GDQS app in terms of correlations with FFQ intakes of fiber, calcium, iron, zinc, and thiamine, and probability of protein and mean micronutrient adequacy, and the AHEI-FFQ was also more strongly correlated with FFQ polyunsaturated fat intake (*P* < 0.05); however, the MDDW-FFQ was also more positively correlated than the GDQS app with energy-adjusted FFQ saturated fat (*P* < 0.05) (Table 4). The GDQS app was more strongly correlated with energy-adjusted FFQ fiber intake than the MDDW-24 and iron than the GDR-24, but the GDR-24 was more strongly correlated with calcium, vitamin A, thiamine, and probability of micronutrient adequacy (*P* < 0.05). The AHEI-24 also outperformed the GDQS app in correlations with energy-adjusted FFQ fiber, iron, and thiamine intakes (*P* < 0.05). As was the case with energy-adjusted probability of protein and mean micronutrient adequacy computed from 24HR data, correlations between these measures computed from FFQ data did not differ between the GDQS-24 and non-GDQS metrics, and the GDQS-FFQ was more strongly correlated than the MDDW-FFQ with energy-adjusted mean probability of micronutrient adequacy computed from the FFQ (*P* < 0.05) (Table 5). The GDQS-FFQ also outperformed the AHEI-FFQ in predicting the probability of protein or mean micronutrient adequacy computed from the FFQ (*P* < 0.05).

**Table 5:** Multivariable associations between Global Diet Quality Score app scores and outcomes related to nutrient adequacy and metabolic risk among Thai adults

Outcome	GDQS app quintile 1	GDQS app quintile 2	GDQS app quintile 3	GDQS app quintile 4	GDQS app quintile 5	Per 1 SD difference in GDQS app	*P*-trend
Continuous outcomes (statistic: estimated marginal mean, 95% CI)							
Probability of protein adequacy computed from 24HR (energy-adjusted), %	63.2 (56.8, 69.5)	67.5 (61.0, 74.0)	66.4 (60.0, 72.9)	69.3 (62.7, 76.0)	72.5 (65.6, 79.4)	3.7 (1.1, 6.2)	0.025∗
Mean probability of micronutrient adequacy computed from 24HR (energy-adjusted), %	36.3 (34.1, 38.5)	38.0 (35.8, 40.2)	39.6 (37.4, 40.6)	40.3 (38.0, 42.6)	44.5 (42.1, 46.9)	2.8 (1.9, 3.7)	<0.001∗
Probability of protein adequacy computed from FFQ (energy-adjusted), %	42.2 (35.4, 48.9)	48.8 (41.8, 55.7)	44.5 (37.7, 51.3)	50.0 (42.8, 57.1)	48.2 (40.9, 55.4)	2.3 (–0.3, 5.0)	0.171
Mean probability of micronutrient adequacy computed from FFQ (energy-adjusted), %	40.6 (36.9, 44.2)	40.6 (36.8, 44.4)	41.9 (38.2, 45.5)	45.78 (41.91, 49.65)	44.2 (40.2, 48.1)	2.1 (0.7, 3.6)	0.018∗
BMI, kg/m^2^	26.3 (25.4, 27.3)	25.8 (24.8, 26.7)	26.0 (25.1, 27.0)	25.8 (24.8, 26.8)	24.9 (23.9, 26.0)	–0.4 (–0.8, 0.0)	0.045∗
MUAC, cm	31.3 (30.5, 32.1)	30.8 (30.0, 31.6)	31.2 (30.4, 32.1)	30.8 (30.0, 31.6)	29.9 (29.1, 30.8)	–0.5 (–0.8, –0.1)	0.017∗
WC, cm	88.8 (86.4, 91.1)	87.6 (85.2, 90.0)	88.7 (86.3, 91.0)	87.0 (84.5, 89.5)	85.5 (83.0, 88.1)	–1.1 (–2.1, –0.2)	0.037∗
Fat mass, %	31.7 (30.3, 33.1)	31.2 (29.8, 32.6)	31.2 (29.8, 32.6)	30.2 (28.8, 31.7)	29.1 (27.6, 30.6)	–0.9 (–1.4, –0.3)	0.002∗
SBP, mm Hg	126 (122, 129)	125 (121, 128)	124 (120, 127)	125 (121, 128)	123 (120, 127)	–1 (–2, 0)	0.355
DBP, mm Hg	84 (82, 87)	83 (81, 85)	82 (80, 84)	82 (79, 84)	81 (79, 84)	–1 (–2, –0)	0.021∗
Hb, g/L	134.3 (131.7, 136.8)	137.5 (134.9, 140.2)	136.3 (133.7, 138.9)	136.4 (133.7, 139.1)	133.4 (130.7, 136.3)	–0.4 (–1.4, 0.7)	0.470
Total cholesterol, mg/dL	211 (202, 219)	217 (208, 226)	209 (200, 217)	205 (196, 214)	206 (197, 215)	–3 (–7, 0)	0.079
LDL cholesterol, mg/dL	147 (139, 155)	147 (138, 155)	146 (138, 155)	141 (132, 150)	140 (131, 149)	–3 (–7, 0)	0.087
HDL cholesterol, mg/dL	49 (47, 52)	51 (48, 53)	49 (46, 51)	51 (49, 54)	53 (50,55)	1 (0, 2)	0.032∗
TG, mg/dL	156 (133, 179)	170 (147, 194)	143 (120, 167)	138 (113, 162)	146 (121, 171)	–10 (–19, –0)	0.113
FPG, mg/dL	113 (106, 119)	111 (104, 117)	116 (110, 123)	110 (103, 117)	111 ( 104, 118)	–2 (–4, 1)	0.743
HbA1C, %	6.0 (5.8, 6.3)	5.9 (5.7, 6.2)	6.2 (5.9, 6.5)	6.1 (5.9, 6.4)	6.0 (5.7, 6.3)	0.0 (–0.2, 0.1)	0.892
Number of MetS components (range: 0–5)	2.5 (2.2, 2.7)	2.4 (2.1, 2.7)	2.4 (2.1, 2.7)	2.4 (2.1, 2.7)	2.1 (1.8, 2.4)	–0.2 (–0.3, 0.0)	0.063
24-h urinary sodium, mg	3560 (3220, 3903)	3623 (3268, 3977)	3639 (3284, 3993)	3567 (3209, 3926)	3703 (3340, 4066)	69 (–69, 212)	0.649
24-h urinary potassium, mg	1385 (1252, 1513)	1396 (1260, 1529)	1404 (1271, 1541)	1560 (1424, 1700)	1646 (1509, 1786)	117 (62, 172)	<0.001∗
Sodium screener score (range: 0–100)	27.5 (25.8, 29.2)	26.9 (25.2, 28.7)	27.8 (26.1, 29.6)	25.8 (23.9, 27.6)	24.0 (22.1, 25.9)	–1.4 (–2.1, –0.7)	0.001∗
Binary outcomes (statistic: multivariable odds ratio, 95%CI)							
BMI ≥25 kg/m^2^	REFERENCE	0.87 (0.52, 1.45)	0.96 (0.58, 1.61)	0.80 (0.47, 1.34)	0.66 (0.39, 1.12)	0.89 (0.75, 1.05)	0.128
High MUAC	REFERENCE	0.88 (0.53, 1.47)	0.98 (0.58, 1.64)	0.78 (0.46, 1.31)	0.52 (0.31, 0.89)	0.83 (0.7, 0.98)	0.020∗
Abdominal obesity	REFERENCE	0.84 (0.50, 1.42)	0.85 (0.50, 1.44)	0.67 (0.39, 1.13)	0.51 (0.30, 0.86)	0.8 (0.67, 0.94)	0.009∗
Waist-to-height ratio >0.5	REFERENCE	1.19 (0.69, 2.07)	1.08 (0.62, 1.89)	0.79 (0.45, 1.36)	0.53 (0.31, 0.92)	0.80 (0.67, 0.96)	0.008∗
Hypertension	REFERENCE	0.99 (0.59, 1.67)	0.67 (0.39, 1.15)	0.83 (0.49, 1.43)	0.70 (0.41, 1.20)	0.87 (0.73, 1.03)	0.154
Anemia	REFERENCE	0.82 (0.43, 1.54)	0.68 (0.34, 1.31)	0.97 (0.51, 1.82)	1.15 (0.62, 2.15)	1.08 (0.88, 1.32)	0.532
Raised LDL cholesterol	REFERENCE	1.33 (0.78, 2.28)	1.27 (0.74, 2.20)	1.02 (0.58, 1.79)	1.09 (0.63, 1.91)	0.96 (0.81, 1.14)	0.899
Reduced HDL cholesterol	REFERENCE	0.68 (0.39, 1.19)	1.19 (0.70, 2.03)	0.92 (0.53, 1.59)	0.66 (0.37, 1.17)	0.93 (0.77, 1.11)	0.419
Raised TG	REFERENCE	0.93 (0.55, 1.58)	0.93 (0.54, 1.58)	1.22 (0.71, 2.08)	0.91 (0.53, 1.57)	0.94 (0.79, 1.12)	0.886
Raised FPG	REFERENCE	1.49 (0.88, 2.52)	1.09 (0.64, 1.85)	1.05 (0.61, 1.80)	0.91 (0.53, 1.57)	0.88 (0.74, 1.04)	0.385
Raised HbA1C	REFERENCE	0.40 (0.14, 1.00)	1.24 (0.58, 2.68)	1.05 (0.47, 2.32)	0.76 (0.32, 1.77)	0.93 (0.70, 1.22)	0.680
MetS	REFERENCE	0.90 (0.53, 1.52)	1.01 (0.60, 1.73)	0.88 (0.51, 1.52)	0.92 (0.53, 1.57)	0.92 (0.77, 1.1)	0.755

Values presented as multivariable estimated marginal means or odds ratios (95% CI) associated with each metric quintile or per 1-SD positive difference in metrics. Models adjusted for age, sex, education, physical activity, smoking, sleep quality, and study group (Mahidol staff compared with community sample). *P*-trend: multivariable *P* for linear trend across metric quintiles. ∗ indicates *P*-trend <0.05.

Abbreviations: BMI, body mass index; CI, confidence interval; DBP, diastolic blood pressure; FFQ, food-frequency questionnaire; FPG, fasting plasma glucose; GDQS, Global Diet Quality Score; Hb, hemoglobin;

Hb, hemoglobin; HbA1C, hemoglobin A1C; HDL, high-density lipoprotein; LDL, low-density lipoprotein; MetS, metabolic syndrome; MUAC, midupper arm circumference; SBP, systolic blood pressure; SD, standard deviation; TG, triglyceride; WC, waist circumference; 24HR, 24-h dietary recall.

### Comparing the performance of the GDQS app, GDQS-24, and GDQS-FFQ in multiple regression models

#### Associations between GDQS app scores compared with assessed outcomes

In males and females, adjusted for age, sex, education, physical activity, smoking, sleep quality, and staff compared with community sampling, GDQS app scores were significantly associated with higher energy-adjusted mean micronutrient adequacy computed from the 24HR (range in estimated marginal mean adequacy between score quintiles 1 and 5: 36.3%–44.5%) and FFQ (Q1–Q5: 40.6%–44.2%) (*P*-trend across quintiles <0.05) (Table 5). The GDQS app was also inversely associated with BMI (Q1–Q5: 26.3–24.9), MUAC (Q1–Q5: 31.3–29.9 cm), WC (Q1–Q5: 88.8–85.5 cm), percentage of fat mass (Q1–Q5: 31.7–29.1%), diastolic BP (Q1–Q5: 84–81 mm Hg), and sodium screener score (Q1–Q5: 27.5–24.0 points); positively associated with HDL cholesterol (Q1–Q5: 49–53 mg/dL) and 24-h urinary potassium (Q1–Q5: 1385–1646 mg); and inversely associated with odds of high MUAC (Q5/Q1 OR: 0.52), abdominal obesity (Q5/Q1 OR: 0.51), and high waist-to-height ratio (Q5/Q1 OR: 0.53) (*P* < 0.05).

#### Comparing associations between GDQS metrics compared with assessed outcomes

Similar to the GDQS app, the GDQS-24 was associated with energy-adjusted mean probability of micronutrient adequacy computed from the 24HR (Q1–Q5: 36.8%–43.4%) and FFQ (Q1–Q5: 38.1%–45.6%) in multivariable models, as was the GDQS-FFQ (Q1–Q5 range in estimated marginal mean adequacy computed from 24HR: 37.1%–41.4%; computed from FFQ: 32.2%–52.5%), whereas the GDQS-FFQ was further associated with energy-adjusted probability of protein adequacy computed from the FFQ (Q1–Q5: 37.7%–51.3%) (*P* < 0.05) (Supplemental Tables 6 and 7). We also observed significant inverse associations between the GDQS-24 and TGs (Q1–Q5 range: 174–142 mg/dL), GDQS-24 and sodium screener score (Q1–Q5: 27.7–24.7 points), and a positive association between the GDQS-FFQ and 24-h urinary potassium (Q1–Q5: 1287–1603 mg) (*P* < 0.05). Associations with energy-adjusted mean probability of micronutrient adequacy computed from the FFQ were significantly stronger for the GDQS-FFQ than both the GDQS app and GDQS-24 (*P* for the difference in trend across quintiles <0.05) but did not otherwise differ for other outcomes (Table 6).

**Table 6:** Statistical comparison of multivariable associations between methods for collecting Global Diet Quality Score (GDQS) data and outcomes related to nutrient adequacy and metabolic risk among Thai adults: GDQS app compared with GDQS-24 compared with GDQS-food-frequency questionnaire

Outcome	GDQS app		GDQS-24		GDQS-FFQ		*P*-diff		
	Per 1 SD	*P*-trend	Per 1 SD	*P*-trend	Per 1 SD	*P*-trend	GDQS app vs. GDQS-24	GDQS app vs. GDQS-FFQ	GDQS-24 vs. GDQS-FFQ
Continuous outcomes (statistic: estimated marginal mean, 95%CI)									
Probability of protein adequacy computed from 24HR (energy-adjusted), %	3.7 (1.1, 6.2)	0.025∗	1.4 (–1.2, 4.0)	0.405	1.6 (–1.0, 4.2)	0.308	0.250	0.344	0.595
Mean probability of micronutrient adequacy computed from 24HR (energy-adjusted), %	2.8 (1.9, 3.7)	<0.001∗	2.3 (1.5, 3.2)	<0.001∗	1.6 (0.7, 2.6)	0.001∗	0.302	0.149	0.297
Probability of protein adequacy computed from FFQ (energy-adjusted), %	2.3 (–0.3, 5.0)	0.171	0.8 (–1.9, 3.6)	0.769	5.5 (2.8, 8.2)	<0.001∗	0.340	0.207	0.155
Mean probability of micronutrient adequacy computed from FFQ (energy-adjusted), %	2.1 (0.7, 3.6)	0.018∗	1.9 (0.5, 3.4)	0.009∗	7.1 (5.8, 8.5)	<0.001∗	0.324	0.014∗	0.028∗
BMI, kg/m^2^	–0.4 (–0.8, 0.0)	0.045∗	–0.1 (–0.5, 0.2)	0.788	–3.4 (–42.0, 35.2)	0.971	0.165	0.158	0.391
MUAC, cm	–0.5 (–0.8, –0.1)	0.017∗	–0.2 (–0.5, 0.1)	0.340	–0.1 (–0.4, 0.3)	0.858	0.193	0.140	0.310
WC, cm	–1.1 (–2.1, –0.2)	0.037∗	–0.6 (–1.5, 0.4)	0.484	–0.4 (–1.4, 0.5)	0.442	0.228	0.259	0.553
Fat mass, %	–0.9 (–1.4, –0.3)	0.002∗	–0.4 (–1.0, 0.2)	0.370	–0.6 (–1.1, 0.0)	0.054	0.141	0.322	0.502
SBP, mm Hg	–1 (–2, 0)	0.355	–1 (–2, 1)	0.686	–1 (–2, 1)	0.960	0.424	0.382	0.644
DBP, mm Hg	–1 (–2, –0)	0.021∗	–1 (–2, 0)	0.127	–1 (–2, 0)	0.580	0.405	0.276	0.427
Hb, g/L	–0.4 (–1.4, 0.7)	0.470	–0.8 (–1.9, 0.2)	0.367	0.3 (–0.8, 1.4)	0.290	0.782	0.338	0.273
Total cholesterol, mg/dL	–3 (–7, 0)	0.079	–2 (–5, 2)	0.339	–3 (–6, 1)	0.076	0.548	0.641	0.388
LDL cholesterol, mg/dL	–3 (–7, 0)	0.087	–2 (–5, 2)	0.469	–2 (–5, 2)	0.121	0.343	0.684	0.384
HDL cholesterol, mg/dL	1 (0, 2)	0.032∗	1 (–0, 2)	0.280	1 (–0, 2)	0.234	0.238	0.427	0.547
TG, mg/dL	–10 (–19, –0)	0.113	–11 (–20, –1)	0.047∗	–9 (–19, 0)	0.204	0.308	0.676	0.362
FPG, mg/dL	–2 (–4, 1)	0.743	–2 (–5, 1)	0.070	1 (–2, 4)	0.264	0.253	0.281	0.167
HbA1C, %	0.0 (–0.2, 0.1)	0.892	–0.1 (–0.2, 0.0)	0.152	0.0 (–0.1, 0.1)	0.630	0.274	0.391	0.233
Number of MetS components (range: 0–5)	–0.2 (–0.3, 0.0)	0.063	–0.1 (–0.2, 0.1)	0.382	–0.1 (–0.2, 0.0)	0.434	0.255	0.463	0.476
24-h urinary sodium, mg	69 (–69, 212)	0.649	–48 (–184, 94)	0.532	–71 (–207, 74)	0.503	0.310	0.476	0.748
24-h urinary potassium, mg	117 (62, 172)	<0.001∗	59 (4, 113)	0.061	98 (43, 156)	<0.001∗	0.260	0.568	0.309
Sodium screener score (range: 0–100)	–1.4 (–2.1, –0.7)	0.001∗	–0.9 (–1.6, –0.2)	0.040∗	–0.1 (–0.8, 0.6)	0.723	0.310	0.100	0.133
Binary outcomes (statistic: multivariable odds ratio, 95%CI)									
BMI ≥25 kg/m^2^	0.89 (0.75, 1.05)	0.128	0.98 (0.83, 1.16)	0.950	0.87 (0.73, 1.03)	0.180	0.217	0.691	0.322
High MUAC	0.83 (0.7, 0.98)	0.020∗	0.94 (0.79, 1.11)	0.553	0.9 (0.75, 1.07)	0.283	0.142	0.265	0.418
Abdominal obesity	0.8 (0.67, 0.94)	0.009∗	0.93 (0.79, 1.1)	0.664	0.9 (0.75, 1.07)	0.200	0.141	0.333	0.439
Waist-to-height ratio >0.5	0.80 (0.67, 0.96)	0.008∗	0.88 (0.73, 1.05)	0.272	0.94 (0.78, 1.12)	0.479	0.239	0.248	0.557
Hypertension	0.87 (0.73, 1.03)	0.154	0.88 (0.74, 1.04)	0.119	0.96 (0.8, 1.14)	0.656	0.990	0.335	0.378
Anemia	1.08 (0.88, 1.32)	0.532	1.13 (0.91, 1.39)	0.412	1.05 (0.85, 1.3)	0.961	0.841	0.649	0.499
Raised LDL cholesterol	0.96 (0.81, 1.14)	0.899	0.90 (0.76, 1.08)	0.451	0.95 (0.79, 1.14)	0.207	0.340	0.322	0.497
Reduced HDL cholesterol	0.93 (0.77, 1.11)	0.419	0.90 (0.75, 1.08)	0.291	0.96 (0.80, 1.16)	0.881	0.444	0.426	0.719
Raised TG	0.94 (0.79, 1.12)	0.886	1.01 (0.85, 1.20)	0.732	0.88 (0.73, 1.05)	0.243	0.450	0.483	0.356
Raised FPG	0.88 (0.74, 1.04)	0.385	1.03 (0.87, 1.23)	0.939	0.95 (0.80, 1.14)	0.621	0.468	0.543	0.398
Raised HbA1C	0.93 (0.70, 1.22)	0.680	0.94 (0.72, 1.23)	0.631	1.08 (0.82, 1.42)	0.407	0.635	0.369	0.343
MetS	0.92 (0.77, 1.1)	0.755	1.06 (0.89, 1.26)	0.424	0.87 (0.72, 1.04)	0.428	0.232	0.390	0.162

Values presented as multivariable estimated marginal means or odds ratios (95% CI) associated with a 1-SD positive difference in metrics. Models adjusted for age, sex, education, physical activity, smoking, sleep quality, and study group (Mahidol staff compared with community sample). *P*-trend: multivariable *P* for linear trend across metric quintiles. *P*-diff: *P* for the difference in linear trends across metric quintiles from the Wald test. ∗ indicates *P*-trend or *P*-diff <0.05.

Abbreviations: BMI, body mass index; CI, confidence interval; DBP, diastolic blood pressure; FFQ / -FFQ, food-frequency questionnaire; FPG, fasting plasma glucose; GDQS, Global Diet Quality Score; Hb, hemoglobin; HbA1C, hemoglobin A1C; HDL, high-density lipoprotein; LDL, low-density lipoprotein; MetS, metabolic syndrome; MUAC, midupper arm circumference; SBP, systolic blood pressure; SD, standard deviation; TG, triglyceride; WC, waist circumference; 24HR / -24, 24-h dietary recall.

### Comparing the performance of the GDQS app, GDQS-24, and GDQS-FFQ with that of the MDDW, AHEI, and GDR scored using 24HR and FFQ data in multiple regression models

#### Comparing associations between GDQS and non-GDQS metrics compared with 24HR and FFQ nutrient intakes

Similar to the GDQS-24, the MDDW-24, AHEI-24, and GDR-24 were positively associated with energy-adjusted mean probability of micronutrient adequacy computed from both the 24HR and the FFQ (*P* < 0.05), whereas none of these metrics were associated with energy-adjusted protein adequacy computed from either instrument (Supplemental Table 8). Similar to the GDQS-FFQ, the MDDW-FFQ and AHEI-FFQ were positively associated with energy-adjusted mean probability of micronutrient adequacy, and protein adequacy computed from the FFQ and the AHEI-FFQ were also associated with the energy-adjusted mean probability of micronutrient adequacy computed from the 24HR (Supplemental Table 9) (*P* < 0.05). For both the GDQS-24 and GDQS-FFQ, associations with energy-adjusted probability of mean micronutrient or protein adequacy did not significantly differ in strength from those observed for other metrics scored using the 24HR and FFQ, respectively (*P >* 0.05). Importantly, performance of the MDDW-24, AHEI-24, and GDR-24 in predicting protein or energy-adjusted mean micronutrient adequacy computed from either the 24HR or FFQ also did not differ from that of the GDQS app, whereas performance of the MDDW-FFQ and AHEI-FFQ in predicting both outcomes computed from the FFQ was stronger than for the GDQS app (*P* < 0.05) (Table 7, Table 8).

**Table 7:** Statistical comparison of multivariable associations between diet metrics and outcomes related to nutrient adequacy and metabolic risk among Thai adults: Global Diet Quality Score (GDQS) app compared with non-GDQS metrics scored using 24-h dietary recall data

Outcome	GDQS app		MDDW-24		AHEI-24		GDR-24		*P*-diff		
	Per 1 SD	*P*-trend	Per 1 SD	*P*-trend	Per 1 SD	*P*-trend	Per 1 SD	*P*-trend	GDQS app vs. MDDW-24	GDQS app vs. AHEI-24	GDQS app vs. GDR-24
Continuous outcomes (statistic: estimated marginal mean, 95%CI)											
Probability of protein adequacy computed from 24HR (energy-adjusted), %	3.7 (1.1, 6.2)	0.025∗	1.5 (–1.0, 4.1)	0.145	0.9 (–1.7, 3.5)	0.352	0.0 (–2.5, 2.6)	0.829	0.391	0.299	0.113
Mean probability of micronutrient adequacy computed from 24HR (energy-adjusted), %	2.8 (1.9, 3.7)	<0.001∗	2.5 (1.6, 3.3)	<0.001∗	1.8 (0.8, 2.7)	0.001∗	2.1 (1.2, 3.0)	<0.001∗	0.441	0.091	0.264
Probability of protein adequacy computed from FFQ (energy-adjusted), %	2.3 (–0.3, 5.0)	0.171	2.7 (–0.1, 5.4)	0.103	1.8 (–1.0, 4.6)	0.539	1.5 (–1.2, 4.2)	0.774	0.532	0.534	0.306
Mean probability of micronutrient adequacy computed from FFQ (energy-adjusted), %	2.1 (0.7, 3.6)	0.018∗	2.6 (1.1, 4.1)	0.002∗	3.1 (1.6, 4.6)	0.002∗	3.3 (1.8, 4.7)	<0.001∗	0.292	0.285	0.127
BMI, kg/m^2^	–0.4 (–0.8, 0.0)	0.045∗	0.1 (–0.3, 0.5)	0.741	–0.3 (–0.7, 0.1)	0.369	–0.1 (–0.5, 0.3)	0.358	0.164	0.334	0.181
MUAC, cm	–0.5 (–0.8, –0.1)	0.017∗	–0.0 (–0.4, 0.3)	0.317	–0.2 (–0.5, 0.2)	0.511	–0.2 (–0.5, 0.2)	0.105	0.192	0.216	0.208
WC, cm	–1.1 (–2.1, –0.2)	0.037∗	0.2 (–0.7, 1.2)	0.678	–0.8 (–1.8, 0.1)	0.178	–0.5 (–1.4, 0.5)	0.199	0.199	0.480	0.248
Fat mass, %	–0.9 (–1.4, –0.3)	0.002∗	–0.0 (–0.6, 0.5)	0.425	–0.8 (–1.4, –0.2)	0.024∗	–0.5 (–1.1, 0.0)	0.028∗	0.138	0.466	0.299
SBP, mm Hg	–1 (–2, 0)	0.355	–0.5 (–1.9, 0.8)	0.402	–0.5 (–1.9, 0.9)	0.857	–0.4 (–1.7, 1.0)	0.484	0.555	0.405	0.530
DBP, mm Hg	–1 (–2, –0)	0.021∗	–0.5 (–1.38, 0.5)	0.293	–0.6 (–1.6, 0.3)	0.310	–0.7 (–1.6, 0.2)	0.201	0.306	0.327	0.391
Hb, g/L	–0.4 (–1.4, 0.7)	0.470	–0.1 (–0.2, 0.1)	0.106	0.0 (–0.1, 0.1)	0.634	0.0 (–0.1, 0.1)	0.688	0.370	0.233	0.222
Total cholesterol, mg/dL	–3 (–7, 0)	0.079	–4 (–8, –1)	0.009∗	–2 (–6, 1)	0.223	–4 (–8, –1)	0.021∗	0.304	0.608	0.224
LDL cholesterol, mg/dL	–3 (–7, 0)	0.087	–4 (–7, –1)	0.012∗	–2 (–5, 1)	0.222	–3 (–7, –0)	0.074	0.413	0.966	0.464
HDL cholesterol, mg/dL	1 (0, 2)	0.032∗	1 (–0, 2)	0.089	1 (0, 2)	0.023∗	0 (–1, 1)	0.990	0.537	0.51	0.132
TG, mg/dL	–10 (–19, –0)	0.113	–4 (–13, 5)	0.210	–12 (–22, –3)	0.020∗	–6 (–16, 3)	0.290	0.688	0.252	0.584
FPG, mg/dL	–1 (–4, 1)	0.743	–3 (–6, –0)	0.042∗	–2 (–5, 1)	0.263	–2 (–5, 1)	0.301	0.242	0.369	0.389
HbA1C, %	0.0 (–0.2, 0.1)	0.892	–0.1 (–0.2, 0.0)	0.138	–0.1 (–0.2, 0.0)	0.281	–0.1 (–0.2, 0.0)	0.112	0.332	0.283	0.273
Number of MetS components (range: 0–5)	–0.2 (–0.3, 0.0)	0.063	–0.1 (–0.1, 0.1)	0.195	–0.1 (–0.2, 0.0)	0.17	–0.0 (–0.2, 0.1)	0.606	0.406	0.603	0.199
24-h urinary sodium, mg	69 (–69, 212)	0.649	58 (–69, 193)	0.569	–30 (–161, 113)	0.879	–101 (–230, 35)	0.311	0.729	0.302	0.210
24-h urinary potassium, mg	117 (62, 172)	<0.001∗	47 (0, 101)	0.162	144 (90, 199)	<0.001∗	86 (31, 137)	0.003∗	0.245	0.365	0.434
Sodium screener score (range: 0–100)	–1.4 (–2.1, –0.7)	0.001∗	–0.5 (–1.2, 0.2)	0.292	–3.8 (–4.4, –3.1)	<0.001∗	–2.1 (–2.8, –1.4)	<0.001∗	0.129	0.012∗	0.106
Binary outcomes (statistic: multivariable odds ratio, 95%CI)											
BMI ≥25 kg/m^2^	0.89 (0.75, 1.05)	0.128	1.04 (0.88, 1.22)	0.514	0.89 (0.75, 1.06)	0.265	0.97 (0.82, 1.14)	0.562	0.334	0.528	0.227
High MUAC	0.83 (0.7, 0.98)	0.020∗	0.9 (0.76, 1.07)	0.023∗	0.94 (0.79, 1.12)	0.902	0.92 (0.78, 1.09)	0.267	0.583	0.165	0.169
Abdominal obesity	0.8 (0.67, 0.94)	0.009∗	1.00 (0.85, 1.19)	0.303	0.86 (0.72, 1.03)	0.064	0.92 (0.78, 1.09)	0.166	0.184	0.466	0.173
Waist-to-height ratio >0.5	0.80 (0.67, 0.96)	0.008∗	0.94 (0.79, 1.11)	0.096	0.81 (0.68, 0.97)	0.009∗	0.88 (0.74, 1.05)	0.034∗	0.296	0.885	0.309
Hypertension	0.87 (0.73, 1.03)	0.154	0.92 (0.78, 1.1)	0.28	0.98 (0.82, 1.17)	0.827	0.95 (0.8, 1.12)	0.326	0.630	0.394	0.615
Anemia	1.08 (0.88, 1.32)	0.532	0.97 (0.78, 1.2)	0.881	0.99 (0.8, 1.22)	0.977	0.95 (0.78, 1.17)	0.584	0.645	0.340	0.268
Raised LDL cholesterol	0.96 (0.81, 1.14)	0.899	0.85 (0.71, 1.02)	0.118	1.00 (0.84, 1.20)	0.559	0.88 (0.74, 1.05)	0.139	0.217	0.376	0.171
Reduced HDL cholesterol	0.93 (0.77, 1.11)	0.419	0.97 (0.81, 1.16)	0.641	0.91 (0.76, 1.10)	0.234	1.06 (0.89, 1.27)	0.325	0.468	0.574	0.118
Raised TG	0.94 (0.79, 1.12)	0.886	0.97 (0.82, 1.15)	0.670	0.96 (0.80, 1.14)	0.791	0.98 (0.83, 1.17)	0.528	0.743	0.935	0.490
Raised FPG	0.88 (0.74, 1.04)	0.385	0.91 (0.77, 1.08)	0.389	0.87 (0.73, 1.04)	0.246	0.94 (0.79, 1.11)	0.462	0.573	0.399	0.925
Raised HbA1C	0.93 (0.70, 1.22)	0.680	0.86 (0.65, 1.13)	0.444	0.98 (0.73, 1.29)	0.939	0.92 (0.70, 1.20)	0.414	0.537	0.728	0.731
MetS	0.92 (0.77, 1.1)	0.755	1.03 (0.86, 1.22)	0.737	0.92 (0.77, 1.11)	0.466	1.02 (0.86, 1.22)	0.546	0.983	0.396	0.292

Values presented as multivariable estimated marginal means or odds ratios (95% CI) associated with a 1-SD positive difference in metrics. Models adjusted for age, sex, education, physical activity, smoking, sleep quality, and study group (Mahidol staff compared with community sample). *P*-trend: multivariable *P* for linear trend across metric quintiles. *P*-diff: *P* for the difference in linear trends across metric quintiles from the Wald test. ∗ indicates *P*-trend or *P*-diff <0.05.

Abbreviations: AHEI, Alternative Healthy Eating Index; BMI, body mass index; DBP, diastolic blood pressure; FFQ / -FFQ, food-frequency questionnaire; FPG, fasting plasma glucose; GDQS, Global Diet Quality Score; GDR, Global Dietary Recommendations; Hb, hemoglobin; HbA1C, hemoglobin A1C; HDL, high-density lipoprotein; LDL, low-density lipoprotein; MDDW, Minimum Dietary Diversity–Women; MetS, metabolic syndrome; MUAC, midupper arm circumference; SBP, systolic blood pressure; TG, triglyceride; WC, waist circumference; 24HR / -24, 24-h dietary recall.

**Table 8:** Statistical comparison of multivariable associations between diet metrics and outcomes related to nutrient adequacy and metabolic risk among Thai adults: Global Diet Quality Score (GDQS) app compared with non-GDQS metrics scored using food-frequency questionnaires data

Outcome	GDQS app		MDDW-FFQ		AHEI-FFQ		*P*-diff^2^	
	Per 1 SD	*P*-trend	Per 1 SD	*P*-trend	Per 1 SD	*P*-trend	GDQS app vs. MDDW-FFQ	GDQS app vs. AHEI-FFQ
Continuous outcomes (statistic: estimated marginal mean, 95%CI)								
Probability of protein adequacy computed from 24HR (energy-adjusted), %	3.7 (1.1, 6.2)	0.025∗	1.8 (–0.8, 4.4)	0.464	1.5 (–1.2, 4.2)	0.407	0.282	0.363
Mean probability of micronutrient adequacy computed from 24HR (energy-adjusted), %	2.8 (1.9, 3.7)	<0.001∗	1.2 (0.3, 2.1)	0.251	1.4 (0.5, 2.4)	<0.001∗	0.164	0.067
Probability of protein adequacy computed from FFQ (energy-adjusted), %	2.3 (–0.3, 5.0)	0.171	4.6 (1.9, 7.3)	0.029	4.0 (1.2, 6.8)	0.013∗	0.348	0.302
Mean probability of micronutrient adequacy computed from FFQ (energy-adjusted), %	2.1 (0.7, 3.6)	0.018∗	6.0 (4.6, 7.4)	<0.001∗	6.2 (4.8, 7.7)	<0.001∗	0.041∗	0.046∗
BMI, kg/m^2^	–0.4 (–0.8, 0.0)	0.045∗	–0.2 (–0.6, 0.2)	0.940	–0.3 (–0.7, 0.1)	0.074	0.361	0.254
MUAC, cm	–0.5 (–0.8, –0.1)	0.017∗	–0.2 (–0.5, 0.2)	0.952	–0.2 (–0.5, 0.1)	0.179	0.216	0.223
WC, cm	–1.1 (–2.1, –0.2)	0.037∗	–0.7 (–1.7, 0.2)	0.722	–1.2 (–2.2, –0.2)	0.011∗	0.817	0.293
Fat mass, %	–0.9 (–1.4, –0.3)	0.002∗	–0.6 (–1.2, –0.0)	0.332	–1.1 (–1.6, –0.5)	<0.001∗	0.639	0.252
SBP, mm Hg	–1 (–2, 0)	0.355	–0.9 (–2.2, 0.5)	0.136	–0.9 (–2.3, 0.5)	0.181	0.645	0.666
DBP, mm Hg	–1 (–2, –0)	0.021∗	–0.9 (–1.9, –0.0)	0.060	–1.0 (–2.0, –0.0)	0.086	0.329	0.557
Hb, g/L	–0.4 (–1.4, 0.7)	0.470	0.0 (–0.1, 0.1)	0.863	0.1 (–0.0, 0.2)	0.100	0.167	0.516
Total cholesterol, mg/dL	–3 (–7, 0)	0.079	–3 (–6, 1)	0.400	–3 (–7, 0)	0.208	0.738	0.571
LDL cholesterol, mg/dL	–3 (–7, 0)	0.087	–1 (–5, 2)	0.739	–2 (–6, 1)	0.278	0.475	0.345
HDL cholesterol, mg/dL	1 (0, 2)	0.032∗	1 (–0, 2)	0.274	1 (0, 2)	0.032∗	0.650	0.437
TG, mg/dL	–10 (–19, –0)	0.113	–14 (–24, –5)	0.078	–14 (–24, –4)	0.012∗	0.214	0.418
FPG, mg/dL	–2 (–4, 1)	0.743	2 (–1, 5)	0.072	–1 (–4, 2)	0.272	0.439	0.207
HbA1C, %	0.0 (–0.2, 0.1)	0.892	0.1 (–0.1, 0.2)	0.227	–0.1 (–0.2, 0.0)	0.081	0.279	0.278
Number of MetS components (range: 0-5)	–0.2 (–0.3, 0.0)	0.063	–0.1 (–0.3, –0.0)	0.129	–0.1 (–0.3, –0.0)	0.012∗	0.584	0.599
24-h urinary sodium, mg	69 (–69, 212)	0.649	–71 (–207, 71)	0.289	–106 (–253, 48)	0.058	0.181	0.334
24-h urinary potassium, mg	117 (62, 172)	<0.001∗	70 (12, 125)	0.093	156 (101, 215)	<0.001∗	0.184	0.272
Sodium screener score (range: 0–100)	–1.4 (–2.1, –0.7)	0.001∗	0.5 (–0.2, 1.2)	0.004∗	–3.4 (–4.1, –2.7)	<0.001∗	0.031∗	0.041∗
Binary outcomes (statistic: multivariable odds ratio, 95% CI)								
BMI ≥25 kg/m^2^	0.89 (0.75, 1.05)	0.128	0.78 (0.65, 0.92)	0.102	0.81 (0.68, 0.97)	0.010∗	0.464	0.523
High MUAC	0.83 (0.7, 0.98)	0.020∗	0.9 (0.76, 1.07)	0.560	0.88 (0.73, 1.05)	0.083	0.298	0.271
Abdominal obesity	0.8 (0.67, 0.94)	0.009∗	0.8 (0.67, 0.96)	0.246	0.8 (0.67, 0.96)	0.004∗	0.775	0.327
Waist-to-height ratio >0.5	0.80 (0.67, 0.96)	0.008∗	0.84 (0.70, 1.01)	0.419	0.78 (0.65, 0.94)	0.007∗	0.352	0.655
Hypertension	0.87 (0.73, 1.03)	0.154	0.85 (0.71, 1.01)	0.085	0.96 (0.8, 1.15)	0.575	0.278	0.613
Anemia	1.08 (0.88, 1.32)	0.532	1.02 (0.82, 1.27)	0.447	1 (0.8, 1.25)	0.740	0.291	0.828
Raised LDL cholesterol	0.96 (0.81, 1.14)	0.899	0.95 (0.79, 1.13)	0.963	0.88 (0.73, 1.06)	0.223	0.614	0.367
Reduced HDL cholesterol	0.93 (0.77, 1.11)	0.419	0.98 (0.82, 1.17)	0.920	0.89 (0.74, 1.08)	0.218	0.352	0.815
Raised TG	0.94 (0.79, 1.12)	0.886	0.88 (0.74, 1.05)	0.219	0.82 (0.68, 0.99)	0.019∗	0.398	0.177
Raised FPG	0.88 (0.74, 1.04)	0.385	0.96 (0.81, 1.15)	0.553	0.94 (0.78, 1.12)	0.583	0.989	0.649
Raised HbA1C	0.93 (0.70, 1.22)	0.680	1.06 (0.81, 1.39)	0.352	0.95 (0.71, 1.26)	0.862	0.337	0.762
MetS	0.92 (0.77, 1.1)	0.755	0.8 (0.67, 0.95)	0.061	0.8 (0.66, 0.96)	0.013∗	0.200	0.278

Values presented as multivariable estimated marginal means or odds ratios (95% CI) associated with a 1-SD positive difference in metrics. Models adjusted for age, sex, education, physical activity, smoking, sleep quality, and study group (Mahidol staff compared with community sample). *P*-trend: multivariable *P* for linear trend across metric quintiles. *P*-diff: *P* for the difference in linear trends across metric quintiles from the Wald test. ∗ indicates *P*-trend or *P*-diff <0.05.

Abbreviations: AHEI, Alternative Healthy Eating Index; BMI, body mass index; CI, confidence interval; DPB, diastolic blood pressure; FFQ / -FFQ, food-frequency questionnaire; FPG, fasting plasma glucose; GDQS, Global Diet Quality Score; Hb, hemoglobin; HbA1C, hemoglobin A1C; HDL, high-density lipoprotein; LDL, low-density lipoprotein; MDDW, Minimum Dietary Diversity–Women; MetS, metabolic syndrome; MUAC, midupper arm circumference; SBP, systolic blood pressure; SD, standard deviation; TG, triglyceride; WC, waist circumference.

#### Comparing associations between GDQS and non-GDQS metrics compared with metabolic outcomes

Metrics scored using 24HR data were varyingly associated with outcomes reflective of metabolic risk: the GDQS-24 was inversely associated with TGs and sodium screener score; the MDDW-24 inversely associated with total and LDL cholesterol, glucose, and odds of high MUAC; the AHEI-24 inversely associated with waist-to-height ratio, percentage of fat mass, TGs, and sodium screener score, and positively associated with HDL cholesterol and 24-h urinary potassium; and the GDR-24 inversely associated with waist-to-height ratio, percentage of fat mass, total cholesterol, and sodium screener score, and positively associated with 24-h urinary potassium (*P* < 0.05) (Supplemental Table 8). In analysis of FFQ data, the GDQS-FFQ and MDDW-FFQ were significantly associated only with higher 24-h urinary potassium and sodium screener scores, whereas numerous associations were observed between the AHEI-FFQ and metabolic outcomes (*P* < 0.05): positive associations with HDL cholesterol and 24h urinary potassium, and inverse associations with WC, waist-to-height ratio, percentage of fat mass, TGs, number of MetS components, sodium screener score and odds of overweight or obesity (BMI ≥25), abdominal obesity, and MetS (a 1 SD positive difference in AHEI-FFQ was associated with an OR of MetS of 0.80), respectively (Supplemental Table 9). The AHEI-24 and AHEI-FFQ exhibited stronger associations with sodium screener score than did the GDQS-24 and GDQS-FFQ (Supplemental Tables 8 and 9), whereas the GDQS app outperformed the MDDW-FFQ and was outperformed by the AHEI-FFQ in predicting sodium screener score, respectively (*P* < 0.05) (Table 8).

## Discussion

This study involved the first use of the GDQS app in a population survey and the first use of the GDQS metric in Southeast Asia. The GDQS app generally outperformed the GDQS and other metrics in terms of correlations with energy-adjusted nutrient intakes computed from the 24HR and FFQ and associations with clinical outcomes. One exception was the superior performance of the AHEI-FFQ in predicting metabolic outcomes, attributable to the metric’s inclusion of key nutrient components (*trans* fat, n–3 fats, polyunsaturated fat, sodium) and the utility of the extended FFQ reference period in capturing metabolic risks given their relatively long latency periods. As expected, metrics computed from 24HR and FFQ data tended to correlate better with energy-adjusted nutrients computed from the 24HR and FFQ, respectively.

With a few exceptions, correlations between the GDQS (computed using the application, 24HR, and FFQ) and energy-adjusted 24HR and FFQ intakes of calcium, fiber, iron, polyunsaturated fat, vitamin A, zinc, and overall nutrient adequacy were significant and positive, as has previously been observed in secondary analyses of data from nonpregnant, nonlactating females in China [31], India [61], Mexico [62], and 10 Sub-Saharan African Countries [63,64]. Negative associations between the GDQS app (although not the GDQS-24 or GDQS-FFQ) and NCD-related outcomes were also generally in line with the GDQS’ performance in prior analyses of cross-sectional data from China and Mexico [31,62] and cohort data from Mexico and the United States [[65], [66], [67]]. However, neither the GDQS nor other metrics in the current study were associated with anemia, as was observed for the GDQS and MDDW in prior analyses of African populations [63,64], possibly because of relatively adequate Hb concentrations in the current population. Also, in contrast with prior cross-sectional analyses [31,[61], [62], [63], [64]], the GDQS app, GDQS-24, and GDQS-FFQ were weakly or insignificantly correlated with 24HR and FFQ energy-adjusted protein intake in the current study. This may be attributed to relatively high dietary protein adequacy in this population and reduced variation with which to derive correlations, coupled with Thais’ moderately high consumption of red and processed meats, both food groups of which are scored negatively in the GDQS to acknowledge their contributions to NCD risk. Partly because of this negative scoring, the GDQS app (although not the GDQS-24 or GDQS-FFQ) was also desirably, albeit modestly, negatively correlated with 24HR energy-adjusted saturated fat intake, whereas correlations between the GDQS and saturated fat have been inconsistent and, in some cases, positive in prior analyses [31,[61], [62], [63], [64]]. Taken together, results of the current and prior studies reflect not only the comparative performance of diet quality metrics but also the influence of both population dietary patterns and prevailing nutritional deficits on these metrics’ empirical validity, results of which (except in the case of the GDQS app) should further be interpreted in the context of inherent limitations of secondary analysis of dietary data [4].

In comparison with the GDQS app, the poorer performance of the GDQS-FFQ and other FFQ-derived metrics in predicting nutrient adequacy and metabolic risk factors may be explained by the fact that FFQs are not generally designed with the objective of accurately capturing absolute intakes or enumerating specific food groups. Poorer performance of the GDQS and other metrics scored using the 24HR than the application may be attributed to key differences in instrument design despite using the same 24-h reference period. For example, in the GDQS app, foods are matched to GDQS-food groups in real-time during the recall using a precoded dropdown menu with help from the participant, whereas foods in a paper-based 24HR are coded during data analysis without the help of the participant or a user interface, which may reduce the accuracy or completeness with which foods are enumerated (these deficits would expectedly be mitigated were a software-assisted 24HR to be used). Also, unlike the 24HR, the GDQS app is designed to capture specific characteristics of foods to score the metric accurately (including whether deep-fried foods were purchased and the colors of certain foods, i.e., deep orange and dark green), and the application prompts participants to recall the use of sweeteners, which are sometimes forgotten in a typical 24HR. The GDQS app’s ability to enumerate consumed food groups more fully than the 24HR is 1 likely reason why the GDQS app exhibited higher scores than the GDQS-24 despite using the same reference period.

Differences in performance between the GDQS app and metrics scored using the 24HR may also be attributable to differences in portion size estimation methods. Although the application’s physical food group quantity models are relatively simplistic in comparison with the various household implements and scales employed by the 24HR used in this study, it is plausible that the application’s direct assessment of consumed amounts of each GDQS-food group provides a more accurate approach than the 24HR’s assessment of each food comprising each group in that only 1 measurement is subject to error instead of several. To quantify the influence of instrument design on the accuracy of the GDQS app to estimate the consumption of each food group and compute metric scores, research led by the Intake Center for Dietary Assessment is currently ongoing to compare food group intakes measured over 1 d of prospective weighed diet records with those measured in a subsequent GDQS app assessment for the same 24-h reference period.

In addition to the strong observed performance of the GDQS app in capturing nutrient adequacy and NCD-related outcomes, we found that the application had several practical advantages in data collection and analysis. Administering the GDQS app required ∼10–20 min compared with the 24HR (which required ∼30–35 min) and the FFQ (which required 45–60 min). Research assistants reported that collecting GDQS app data was less cognitively burdensome for participants than collecting 24HR or FFQ data, as the application’s onboard database, dropdown menu, user interface, and prompts considerably eased the interview process, whereas the assessment instrument itself (a tablet) and accompanying stackable, lightweight cubes were more portable than the 24HR recall protocol book, measuring cup, household utensils, and scale. Furthermore, although data processing and analysis were relatively laborious for the 24HR and FFQ, analysis of the GDQS app was relatively straightforward as all the preanalytical work (i.e., data entry, matching foods to food groups, and computing metric scores) was performed onboard the device at the point of data collection.

To our knowledge, the GDQS and GDQS app are currently the only validated metrics and tools designed to jointly capture as proxies for dietary risk of nutrient adequacy and diet-related NCD risk in diverse populations, respectively. List-based screeners, which capture consumption of “sentinel” foods within food groups, have been developed for assessing the MDDW and GDR more rapidly than open-recall methods (i.e., in which consumption of all foods is assessed at the level of individual foods), but they employ cruder methods of assessing consumed amounts than the GDQS app (consumption each food group in the DQQ and MDDW is classified as “yes/no” or relative to a 15 g threshold, respectively [25,27,42]). By contrast, the GDQS app is unique in that it is the only tool that uses information from an open dietary recall of individual foods in combination with physical models to guide assessment of amounts of consumption at the level of food groups, an approach which preserves the richness of the underlying food consumption data (which can be very important for guiding policies and programs) while simplifying and (as demonstrated in this study) potentially improving the accuracy of metric computation in comparison with that which could otherwise be achieved in secondary analysis of paper-based 24HR data. Investigators have compared MDDW and GDR scores computed using country-specific list-based screeners and DQQs with open-24HR data to evaluate relationships between the MDDW and GDR compared with other metrics and indices, food group and nutrient intakes, and BMI in different countries [[68], [69], [70]], but have not yet compared metric-outcome associations derived using list-based and open-recall approaches, respectively.

A strength of this study was the expansiveness of data collection, which included numerous outcomes related to nutrient adequacy and metabolic risk, 3 methods of dietary assessment (GDQS app, repeat 24HR, and FFQ), and 8 diet metrics for each participant, which allowed detailed comparisons of the performance of different approaches to scoring diet metrics as well as validation of diet metrics against nutrient intakes and adequacy computed using 24-h and month-long reference periods. Conducting the study in Thailand provided an aptly challenging environment to rigorously evaluate the performance of the GDQS app, given particularly complex aspects of Southeast Asian diets, such as a predominance of mixed and shared dishes. A key limitation of this study was the cross-sectional nature of data collection, which has provided compelling evidence for the GDQS app’s performance but has limited our ability to infer causal relations between diet quality metrics and outcomes, particularly those related to NCD risk. Additional research involving longitudinal assessments will, therefore, be helpful for further understanding the application’s performance, as will research in other age and groups and populations.

In conclusion, this study demonstrates the effectiveness of the GDQS app for understanding population nutrient adequacy and metabolic risks among Thai adults, and superior performance of the application than the GDQS and other metrics scored using 24HR and FFQ data, and thus supports the use of this tool in population surveys. Our results also generally highlight the potential benefits of directly assessing diet metrics using purpose-built instruments instead of scoring them using data from conventional dietary assessment instruments (nonetheless, the 24HR and FFQ remain critical tools in nutrition surveys, particularly for capturing absolute nutrient intakes and estimating associations between long-term diet and disease risk, respectively).

## Author contributions

The authors’ responsibilities were as follows – SB, TP, WWF, PW, WK: conceptualized the study; SB, TP, WWF, WK: led funding acquisition; SB, TP, WWF, PW, WCW, WK: designed the study; MD, MM, JA, NBA: provided essential materials and expertise; TP, AP, PS, WS, SP: collected data; SB, TP, AP, PS: analyzed data; SB, TP, AP, MD, MM, CB, WCW, WK: interpreted results; SB, TP, AP, PS, WK: wrote the paper; SB: had primary responsibility for final content; and all authors: read and approved the final manuscript.

## Conflict of interest

SB reports financial support was provided by National Institutes of Health. TP reports financial support was provided by The Rockefeller Foundation. WK reports financial support was provided by Health Systems Research Institute, Thailand. All other authors report no conflicts of interest.

## Funding

This study was funded by Health Systems Research Institute, Thailand (#64-213); Fogarty International Center, National Institutes of Health (#D43 TW010543); The Rockefeller Foundation (#2021 FOD 024).

## Data availability

To express interest in using the GDQS app, please contact GDQS@FHISolutions.org. To access other tools used in this study and the analytical dataset, please contact the authors.

## Declaration of interests

The authors declare the following financial interests/personal relationships which may be considered as potential competing interests: Sabri Bromage reports financial support was provided by National Institutes of Health. Tippawan Pongcharoen reports financial support was provided by The Rockefeller Foundation. Wantanee Kriengsinyos reports financial support was provided by Health Systems Research Institute (Thailand).
