# Supplementary material for: Performance of the Global Diet Quality Score (GDQS) App in Predicting Nutrient Adequacy and Metabolic Risk Factors among Thai Adults
Source: J Nutr. 2023 Oct 14;153(12):3576–94. doi: 10.1016/j.tjnut.2023.10.007 (PMC10739769; doi:10.1016/j.tjnut.2023.10.007)

**ONLINE SUPPLEMENTARY MATERIAL**

Performance of the Global Diet Quality Score (GDQS) app in predicting nutrient adequacy and metabolic risk factors among Thai adults (Bromage S, et al)

**Supplemental Table 1:** Extent of missing data

| **Measurement** | **Missing data points, % (n)** |
| --- | --- |
| 1 day of GDQS app | 0.2% (1) |
| Metrics (GDQS-24, MDDW-24, AHEI-24, GDR-24) and nutrient intakes computed from 24HR |  |
| Missing day 2 of 24HR | 3.0% (18) |
| Missing days 1 and 2 24HR | 0 |
| Metrics (GDQS-FFQ, MDDW-FFQ, AHEI-FFQ) and nutrient intakes computed from FFQ | 3.0% (18) |
| Sociodemographic questionnaire | 0 |
| Global Physical Activity Questionnaire | 0 |
| Smoking questionnaire | 0 |
| Pittsburgh Sleep Quality Index questionnaire | 0 |
| Sodium intake screener | 0 |
| Duplicate measures of weight, height, and BMI | 0 |
| MUAC | 0 |
| Duplicate measures of midpoint WC | 0 |
| Body composition measurements | 0.2% (1) |
| Triplicate measures of SBP and DBP | 0 |
| Complete blood count (including Hb) | 0.2% (1) |
| Total, HDL, and LDL cholesterol, and TG | 0 |
| FBG | 0 |
| HbA1C | 0.2% (1) |
| Complete 24-hour urine collection for sodium and potassium intake estimation | 32.0% (192) |

**Footnote:** Abbreviations: GDQS, Global Diet Quality Score; MDDW, Minimum Dietary Diversity-Women; AHEI, Alternative Healthy Eating Index; GDR, Global Dietary Recommendations; 24HR / -24, 24-hour dietary recall; FFQ / -FFQ, food-frequency questionnaire; BMI: body-mass index; MUAC, mid-upper arm circumference; WC, waist circumference; SBP, systolic blood pressure; DPB, diastolic blood pressure; Hb, hemoglobin; LDL, low-density lipoprotein; HDL, high-density lipoprotein; TG, triglyceride; FPG, fasting plasma glucose; HbA1C, hemoglobin A1C.

**Supplemental Table 2.** Statistical comparison of mean diet metric scores by data collection tool (GDQS app, 24HR, FFQ) and sex

|  | **GDQS app** | **24HR** | **FFQ** | ***p*-diff** | | |
| --- | --- | --- | --- | --- | --- | --- |
|  |  |  |  | **GDQS app vs 24HR** | **GDQS app vs FFQ** | **24HR vs FFQ** |
| Men |  |  |  |  |  |  |
| GDQS | 19.6 (4.6) | 16.7 (4.1)* | 20.6 (4.9)* | **<0.001** | 0.150 | **<0.001** |
| MDDW | N/A | 4.3 (1.5) | 6.8 (1.6) | N/A | N/A | **<0.001** |
| AHEI-2010 | N/A | 38.6 (10.8) | 51.4 (11.2)* | N/A | N/A | **<0.001** |
| GDR | N/A | 9.0 (1.1)* | N/A | N/A | N/A | N/A |
| Women |  |  |  |  |  |  |
| GDQS | 19.4 (4.9) | 17.9 (3.9)* | 21.6 (5.3)* | **<0.001** | **<0.001** | **<0.001** |
| MDDW | N/A | 4.4 (1.4) | 7.0 (1.5) | N/A | N/A | **<0.001** |
| AHEI-2010 | N/A | 40.6 (11.2) | 55.5 (11.8)* | N/A | N/A | **<0.001** |
| GDR | N/A | 9.5 (2.1)* | N/A | N/A | N/A | N/A |

**Footnote:** Statistics estimated from day 1 of GDQS app and 24HR collection. *p*-diff, p for pairwise difference between instruments. *p-*diff and differences between men and women estimated from ANOVA. Bold cells and * indicate *p-*diff or p for difference between men and women <0.05, respectively. Abbreviations: GDQS, Global Diet Quality Score; MDDW, Minimum Dietary Diversity-Women; AHEI-2010, Alternative Healthy Eating Index-2010; GDR, Global Dietary Recommendations; 24HR, 24hr dietary recall; FFQ, food frequency questionnaire.

**Supplemental Table 3.** Spearman’s correlation between diet metric scores by data collection tools (GDQS app, 24HR, FFQ)

|  | **GDQS app** | **GDQS-24** | **MDDW-24** | **AHEI-24** | **GDR-24** | **GDQS-FFQ** | **MDDW-FFQ** | **AHEI-FFQ** |
| --- | --- | --- | --- | --- | --- | --- | --- | --- |
| **GDQS app** | 1.00 | 0.55 | 0.42 | 0.53 | 0.56 | 0.23 | 0.17 | 0.32 |
| **GDQS-24** |  | 1.00 | 0.52 | 0.53 | 0.55 | 0.21 | 0.14 | 0.29 |
| **MDDW-24** |  |  | 1.00 | 0.32 | 0.37 | 0.19 | 0.18 | 0.21 |
| **AHEI-24** |  |  |  | 1.00 | 0.57 | 0.25 | 0.09 | 0.50 |
| **GDR-24** |  |  |  |  | 1.00 | 0.24 | 0.12 | 0.36 |
| **GDQS-FFQ** |  |  |  |  |  | 1.00 | 0.75 | 0.75 |
| **MDDW-FFQ** |  |  |  |  |  |  | 1.00 | 0.55 |
| **AHEI-FFQ** |  |  |  |  |  |  |  | 1.00 |

**Footnote:** Recall day 1 was used for correlations involving the GDQS app and metrics scored using 24HR data. Dark green shading indicates the strongest observed correlation (0.75) and dark red indicates the weakest (0.09). Abbreviations: GDQS, Global Diet Quality Score; MDDW, Minimum Dietary Diversity-Women; AHEI-2010, Alternative Healthy Eating Index-2010; GDR, Global Dietary Recommendations; 24HR / -24, 24hr dietary recall; FFQ / -FFQ, food frequency questionnaire.

**Supplemental Table 4.** Concordance correlation (95%CI) between the same diet metrics computed using different data collection tools (GDQS app, 24HR, FFQ)

|  | **Instrument** | | |
| --- | --- | --- | --- |
| **Metric** | **24HR vs. FFQ** | **App vs. 24HR** | **App vs. FFQ** |
| GDQS | 0.15 (0.10, 0.21) | 0.49 (0.44, 0.55) | 0.22 (0.14, 0.29) |
| MDDW | 0.07 (0.04, 0.10) | N/A | N/A |
| AHEI-2010 | 0.28 (0.23, 0.32) | N/A | N/A |

**Footnote:** Recall day 1 was used for correlations involving the GDQS app and metrics scored using 24HR data. Abbreviations: GDQS, Global Diet Quality Score; MDDW, Minimum Dietary Diversity-Women; AHEI-2010, Alternative Healthy Eating Index-2010; 24HR, 24hr dietary recall; FFQ, food frequency questionnaire.

**Supplemental Table 5.** Statistical comparison of Spearman correlations between diet metrics and outcomes related to nutrient adequacy and metabolic risk among Thai adults: GDQS-24 vs. non-GDQS metrics scored using 24HR data and GDQS-FFQ vs. non-GDQS metrics scored using FFQ data

| **Outcome** | **Metrics scored using 24HR** | | | | | | | **Metrics scored using FFQ** | | | | |
| --- | --- | --- | --- | --- | --- | --- | --- | --- | --- | --- | --- | --- |
|  | **r_s_ (*P*)** | | | | ***p*-diff** | | | **r_s_ (p)** | | | ***p*-diff** | |
|  | **GDQS-24** | **MDDW-24** | **AHEI-24** | **GDR-24** | **GDQS-24 vs MDDW-24** | **GDQS-24 vs AHEI-24** | **GDQS-24 vs GDR-24** | **GDQS-FFQ** | **MDDW-FFQ** | **AHEI-FFQ** | **GDQS-FFQ vs MDDW-FFQ** | **GDQS-FFQ vs AHEI-FFQ** |
| ***Energy-adjusted 24HR nutrient intakes and adequacy*** | | | | | | | | | | | | |
| Protein | 0.07 (0.097) | 0.07 (0.088) | 0.04 (0.385) | -0.01 (0.752) | 0.953 | 0.317 | 0.042 | 0.12 (**0.004**) | 0.07 (0.098) | 0.07 (0.105) | 0.048 | 0.112 |
| Saturated fat | 0.01 (0.807) | 0.04 (0.362) | -0.05 (0.248) | -0.14 (**<0.001**) | 0.601 | 0.129 | **<0.001** | 0.03 (0.479) | 0.06 (0.166) | -0.05 (0.253) | 0.275 | **0.003** |
| Monounsaturated fat | 0.07 (0.097) | 0.07 (0.090) | 0.05 (0.219) | -0.07 (0.082) | 0.783 | 0.489 | **<0.001** | 0.03 (0.462) | 0.05 (0.259) | -0.02 (0.685) | 0.608 | 0.141 |
| Polyunsaturated fat | 0.11 (**0.005**) | 0.10 (**0.011**) | 0.24 (**<0.001**) | 0.03 (0.436) | 0.520 | **0.001** | **0.012** | 0.10 (**0.025**) | 0.12 (**0.004**) | 0.11 (**0.010**) | 0.393 | 0.244 |
| Fiber | 0.34 (**<0.001**) | 0.23 (**<0.001**) | 0.46 (**<0.001**) | 0.39 (**<0.001**) | **0.004** | **0.001** | 0.332 | 0.26 (**<0.001**) | 0.18 (**<0.001**) | 0.38 (**<0.001**) | **0.007** | **<0.001** |
| Calcium | 0.19 (**<0.001**) | 0.21 (**<0.001**) | 0.24 (**<0.001**) | 0.18 (<0.001) | 0.632 | 0.223 | 0.852 | 0.21 (**<0.001**) | 0.20 (**<0.001**) | 0.25 (**<0.001**) | 0.797 | 0.152 |
| Iron | 0.16 (**<0.001**) | 0.16 (**<0.001**) | 0.10 (**0.010**) | 0.10 (0.011) | 0.998 | 0.179 | 0.166 | 0.14 (**0.001**) | 0.08 (0.063) | 0.13 (**0.001**) | **0.024** | 0.808 |
| Zinc | 0.14 (**0.001**) | 0.06 (0.130) | 0.08 (0.052) | 0.12 (**0.003**) | **0.036** | 0.100 | 0.495 | 0.02 (0.627) | 0.00 (0.867) | 0.03 (0.500) | 0.369 | 0.925 |
| Vitamin A | 0.20 (**<0.001**) | 0.26 (**<0.001**) | 0.19 (**<0.001**) | 0.18 (**<0.001**) | 0.192 | 0.858 | 0.519 | 0.21 (**<0.001**) | 0.16 (**<0.001**) | 0.24 (**<0.001**) | 0.118 | 0.275 |
| Thiamine | 0.15 (**<0.001**) | 0.16 (**<0.001**) | 0.07 (0.081) | 0.11 (**0.008**) | 0.888 | **0.033** | 0.238 | 0.07 (0.077) | 0.05 (0.191) | 0.02 (0.547) | 0.641 | 0.158 |
| Vitamin B12 | 0.13 (**0.002**) | 0.16 (**<0.001**) | 0.03 (0.442) | 0.09 (**0.021**) | 0.620 | 0.012 | 0.413 | 0.07 (0.089) | 0.05 (0.223) | 0.07 (0.104) | 0.400 | 0.647 |
| Probability of protein adequacy | 0.07 (0.082) | 0.06 (0.124) | 0.06 (0.141) | 0.01 (0.735) | 0.679 | 0.826 | 0.265 | 0.07 (0.087) | 0.09 (**0.040**) | 0.10 (**0.016**) | 0.755 | 0.518 |
| Mean probability of micronutrient adequacy | 0.23 (**<0.001**) | 0.25 (**<0.001**) | 0.21 (**<0.001**) | 0.19 (**<0.001**) | 0.709 | 0.571 | 0.322 | 0.18 (**<0.001**) | 0.10 (**0.015**) | 0.20 (**<0.001**) | **0.007** | 0.426 |
| ***Energy-adjusted FFQ nutrient intakes and adequacy*** | | | | | | | | | | | | |
| Protein | 0.04 (0.355) | 0.00 (0.987) | 0.03 (0.520) | 0.02 (0.608) | 0.255 | 0.845 | 0.979 | 0.09 (**0.036**) | 0.02 (0.584) | 0.05 (0.238) | 0.016 | 0.267 |
| Saturated fat | 0.11 (**0.010**) | 0.04 (0.367) | 0.01 (0.751) | -0.06 (0.156) | **0.048** | **0.026** | **<0.001** | 0.07 (0.085) | 0.10 (**0.016**) | 0.02 (0.692) | 0.433 | 0.110 |
| Monounsaturated fat | 0.09 (**0.036**) | 0.04 (0.307) | 0.01 (0.721) | 0.00 (0.973) | 0.213 | 0.064 | 0.069 | 0.04 (0.346) | 0.01 (0.873) | 0.01 (0.758) | 0.244 | 0.520 |
| Polyunsaturated fat | 0.14 (**0.001**) | 0.03 (0.453) | 0.09 (**0.024**) | 0.06 (0.165) | **0.006** | 0.306 | 0.071 | 0.17 (**<0.001**) | 0.09 (**0.026**) | 0.19 (**<0.001**) | **0.009** | 0.300 |
| Fiber | 0.18 (**<0.001**) | 0.12 (**0.004**) | 0.34 (**<0.001**) | 0.27 (**<0.001**) | 0.156 | **<0.001** | 0.068 | 0.49 (<0.001) | 0.33 (**<0.001**) | 0.62 (**<0.001**) | **<0.001** | **<0.001** |
| Calcium | 0.15 (**<0.001**) | 0.13 (**0.002**) | 0.16 (**<0.001**) | 0.19 (**<0.001**) | 0.394 | 0.869 | 0.474 | 0.49 (**<0.001**) | 0.45 (**<0.001**) | 0.45 (**<0.001**) | 0.070 | 0.197 |
| Iron | 0.17 (**<0.001**) | 0.09 (**0.029**) | 0.20 (**<0.001**) | 0.02 (**<0.001**) | 0.038 | 0.552 | 0.250 | 0.38 (<0.001) | 0.26 (**<0.001**) | 0.37 (**<0.001**) | **<0.001** | 0.929 |
| Zinc | 0.10 (**0.015**) | 0.08 (0.059) | 0.08 (0.057) | 0.07 (0.102) | 0.381 | 0.529 | 0.824 | 0.17 (**<0.001**) | 0.11 (**0.008**) | 0.12 (**0.003**) | 0.024 | 0.101 |
| Vitamin A | 0.14 (**0.001**) | 0.11 (**0.007**) | 0.15 (**<0.001**) | 0.20 (**<0.001**) | 0.459 | 0.805 | 0.155 | 0.11 (**0.007**) | 0.04 (0.356) | 0.14 (**<0.001**) | **0.004** | 0.100 |
| Thiamine | 0.12 (**0.004**) | 0.08 (**0.045**) | 0.15 (**<0.001**) | 0.12 (**0.003**) | 0.260 | 0.533 | 0.962 | 0.26 (**<0.001**) | 0.17 (**<0.001**) | 0.27 (**<0.001**) | **0.001** | 0.715 |
| Vitamin B12 | 0.09 (**0.034**) | 0.05 (0.238) | 0.02 (0.703) | 0.10 (**0.020**) | 0.299 | 0.074 | 0.659 | 0.01 (0.789) | -0.01 (0.767) | -0.02 (0.627) | 0.312 | 0.364 |
| Probability of protein adequacy | 0.04 (0.336) | 0.08 (**0.042**) | 0.01 (0.782) | 0.03 (0.515) | 0.569 | 0.345 | 0.830 | 0.21 (**<0.001**) | 0.18 (**<0.001**) | 0.14 (**<0.001**) | 0.190 | **0.005** |
| Mean probability of micronutrient adequacy | 0.16 (**<0.001**) | 0.17 (**<0.001**) | 0.17 (**<0.001**) | 0.19 (**<0.001**) | 0.677 | 0.939 | 0.436 | 0.46 (**<0.001**) | 0.38 (**<0.001**) | 0.39 (**<0.001**) | **0.003** | **0.014** |
| ***Clinical and biochemical measurements*** | | | | | | | | | | | | |
| BMI | -0.07 (0.073) | -0.06 (0.176) | -0.08 (0.054) | -0.04 (0.369) | 0.611 | 0.546 | 0.766 | -0.05 (0.199) | -0.08 (**0.041**) | -0.13 (**0.002**) | 0.231 | **0.025** |
| Fat mass, % | 0.01 (0.798) | -0.02 (0.620) | -0.04 (0.317) | 0.00 (0.972) | 0.308 | 0.076 | 0.335 | -0.04 (0.353) | -0.05 (0.222) | -0.06 (0.147) | 0.333 | 0.517 |
| MUAC | -0.10 (**0.016**) | -0.07 (0.090) | -0.07 (0.075) | -0.06 (0.137) | 0.383 | 0.847 | 0.738 | -0.07 (0.094) | -0.09 (**0.030**) | -0.13 (**0.001**) | 0.439 | 0.083 |
| WC | -0.10 (**0.013**) | -0.05 (0.215) | -0.09 (**0.033**) | -0.06 (0.123) | 0.196 | 0.972 | 0.650 | -0.09 (**0.039**) | -0.10 (**0.012**) | -0.17 (**<0.001**) | 0.494 | **0.009** |
| SBP | -0.05 (0.192) | -0.04 (0.301) | 0.02 (0.660) | -0.02 (0.576) | 0.583 | 0.119 | 0.419 | -0.04 (0.280) | -0.09 (**0.024**) | -0.07 (0.111) | 0.141 | 0.638 |
| DBP | -0.10 (**0.017**) | -0.06 (0.178) | -0.04 (0.277) | -0.08 (0.060) | 0.251 | 0.235 | 0.584 | -0.08 (0.071) | -0.12 (**0.004**) | -0.11 (**0.009**) | 0.170 | 0.407 |
| Hb | -0.09 (**0.029**) | -0.05 (0.250) | -0.03 (0.479) | -0.03 (0.476) | 0.169 | 0.138 | 0.149 | -0.04 (0.286) | -0.06 (0.185) | -0.05 (0.200) | 0.957 | 0.845 |
| Total cholesterol | 0.00 (0.984) | -0.07 (0.106) | 0.03 (0.517) | -0.07 (0.075) | 0.071 | 0.737 | 0.091 | -0.04 (0.295) | -0.04 (0.371) | -0.01 (0.837) | 0.984 | 0.362 |
| LDL-C | -0.02 (0.623) | -0.08 (0.061) | -0.02 (0.657) | -0.07 (0.083) | 0.181 | 0.795 | 0.257 | -0.03 (0.543) | -0.02 (0.635) | -0.02 (0.577) | 0.919 | 0.745 |
| HDL-C | 0.09 (**0.023**) | 0.08 (**0.038**) | 0.12 (**0.003**) | 0.06 (0.165) | 0.640 | 0.547 | 0.617 | 0.09 (**0.031**) | 0.08 (**0.041**) | 0.16 (**<0.001**) | 0.971 | 0.028 |
| TG | -0.08 (0.055) | -0.07 (0.078) | -0.08 (**0.041**) | -0.09 (**0.020**) | 0.876 | 0.548 | 0.391 | -0.08 (0.060) | -0.09 (**0.038**) | -0.16 (**<0.001**) | 0.441 | **0.007** |
| FPG | -0.08 (0.060) | -0.08 (**0.048**) | -0.07 (0.090) | -0.06 (0.134) | 0.908 | 0.936 | 0.952 | -0.02 (0.582) | -0.02 (0.641) | -0.06 (0.158) | 0.916 | 0.340 |
| HbA1C | 0.02 (0.671) | 0.01 (0.825) | 0.03 (0.529) | -0.02 (0.667) | 0.890 | 0.776 | 0.205 | 0.00 (0.960) | 0.01 (0.863) | -0.03 (0.419) | 0.975 | 0.232 |
| Number of MetS components | -0.05 (0.182) | -0.07 (0.100) | -0.04 (0.361) | 0.00 (0.895) | 0.844 | 0.988 | 0.359 | -0.07 (0.077) | -0.11 (**0.007**) | -0.12 (**0.003**) | 0.135 | 0.259 |
| 24-hour urinary sodium | -0.07 (0.153) | 0.02 (0.680) | -0.07 (0.166) | -0.08 (0.098) | 0.069 | 0.956 | 0.878 | -0.06 (0.256) | -0.03 (0.512) | -0.14 (**0.004**) | 0.523 | 0.014 |
| 24-hour urinary potassium | 0.12 (**0.017**) | 0.13 (**0.009**) | 0.22 (**<0.001**) | 0.13 (**0.010**) | 0.758 | 0.102 | 0.651 | 0.21 (2.493) | 0.13 (**0.008**) | 0.26 (**<0.001**) | 0.088 | 0.173 |
| Sodium screener score | -0.15 (**<0.001**) | -0.11 (**0.010**) | -0.47 (**<0.001**) | -0.23 (**<0.001**) | 0.361 | **<0.001** | 0.058 | -0.08 (**0.047**) | 0.02 (0.562) | -0.45 (**<0.001**) | **0.001** | **<0.001** |

**Footnote:** *p*-diff: *p* for difference estimated using Wolfe’s test for dependent correlation coefficients. Bold cells indicate *p* for significance of correlation coefficient <0.05 or *p-*diff <0.05. Abbreviations: GDQS, Global Diet Quality Score; MDDW, Minimum Dietary Diversity-Women; AHEI, Alternative Healthy Eating Index; GDR, Global Dietary Recommendations; 24HR / -24, 24-hour dietary recall; FFQ / -FFQ, food-frequency questionnaire; BMI: body-mass index; MUAC, mid-upper arm circumference; WC, waist circumference; SBP, systolic blood pressure; DPB, diastolic blood pressure; Hb, hemoglobin; LDL, low-density lipoprotein; HDL, high-density lipoprotein; TG, triglyceride; FPG, fasting plasma glucose; HbA1C, hemoglobin A1C; MetS, metabolic syndrome.

**Supplemental Table 6.** Multivariable associations between the GDQS-24 and outcomes related to nutrient adequacy and metabolic risk among Thai adults

| **Outcome** | **GDQS-24 quintile 1** | **GDQS-24 quintile 2** | **GDQS-24 quintile 3** | **GDQS-24 quintile 4** | **GDQS-24 quintile 5** | **Per 1 SD difference in GDQS-24** | ***p*-trend** |
| --- | --- | --- | --- | --- | --- | --- | --- |
| ***Continuous outcomes (statistic: estimated marginal mean, 95%CI)*** | | | | | | | |
| Probability of protein adequacy computed from 24HR (energy-adjusted), % | 65.2 (58.9, 71.5) | 70.7 (64.5, 76.8) | 63.2 (56.5, 70.0) | 67.8 (61.0, 74.6) | 70.5 (63.5, 77.4) | 1.4 (-1.2, 4.0) | 0.405 |
| Mean probability of micronutrient adequacy computed from 24HR (energy-adjusted), % | 36.8 (34.6, 39.0) | 38.4 (36.3, 40.5) | 39.4 (37.0, 41.7) | 41.2 (38.9, 43.6) | 43.4 (41.0, 45.8) | 2.3 (1.5, 3.2) | **<0.001** |
| Probability of protein adequacy computed from FFQ (energy-adjusted), % | 44.1 (37.3, 50.8) | 50.2 (43.6, 56.7) | 43.7 (36.4, 51.0) | 46.4 (39.1, 53.6) | 47.4 (39.9, 54.8) | 0.8 (-1.9, 3.6) | 0.769 |
| Mean probability of micronutrient adequacy computed from FFQ (energy-adjusted), % | 38.1 (34.5, 41.7) | 44.4 (40.9, 47.9) | 42.6 (38.7, 46.5) | 43.2 (39.3, 47.03) | 45.6 (41.6, 49.6) | 1.9 (0.5, 3.4) | **0.009** |
| Body-mass index, kg/m^2^ | 25.8 (24.8, 26.7) | 25.6 (24.7, 26.6) | 26.4 (25.4, 27.4) | 25.9 (24.9, 26.9) | 25.5 (24.4, 26.5) | -0.1 (-0.5, 0.2) | 0.788 |
| Mid-upper arm circumference, cm | 30.9 (30.1, 31.7) | 30.7 (30.0, 31.5) | 31.5 (30.7, 32.4) | 30.7 (29.8, 31.6) | 30.4 (29.5, 31.3) | -0.2 (-0.5, 0.1) | 0.340 |
| Waist circumference, cm | 88.1 (85.8, 90.5) | 87.1 (84.8, 89.4) | 88.8 (86.2, 91.3) | 87.3 (84.8, 89.8) | 86.9 (84.3, 89.4) | -0.6 (-1.5, 0.4) | 0.484 |
| Fat mass, % | 31.0 (29.6, 32.4) | 30.6 (29.3, 32.0) | 31.5 (30.0, 33.0) | 30.7 (29.3, 32.2) | 30.0 (28.5, 31.5) | -0.4 (-1.0, 0.2) | 0.370 |
| Systolic blood pressure, mmHg | 126 (122, 129) | 123 (120, 127) | 125 (122, 129) | 123 (120, 127) | 125 (121, 128) | -1 (-2, 1) | 0.686 |
| Diastolic blood pressure, mmHg | 84 (82, 86) | 82 (79, 84) | 84 (81, 86) | 81 (78, 83) | 82 (80, 85) | -1 (-2, 0) | 0.127 |
| Hemoglobin, g/L | 135.8 (133.2, 138.4) | 136.6 (134.1 139.1) | 135.6 (132.9, 138.4) | 135.0 (132.2, 137.7) | 134.9 (132.1, 137.8) | -0.8 (-1.9, 0.2) | 0.367 |
| Total cholesterol, mg/dL | 206 (198, 214) | 218 (210, 226) | 206 (196, 215) | 213 (204, 222) | 202 (193, 212) | -2 (-5, 2) | 0.339 |
| LDL-C, mg/dL | 142 (133, 150) | 151 (143, 159) | 143 (134, 152) | 147 (138, 155) | 139 (130, 148) | -2 (-5, 2) | 0.469 |
| HDL-C, mg/dL | 49 (47, 52) | 51 (49, 53) | 49 (46, 51) | 53 (50, 55) | 50 (48, 53) | 1 (-0, 2) | 0.280 |
| Triglyceride, mg/dL | 174 (151, 197) | 142 (119, 164) | 152 (127, 177) | 139 (115, 165) | 142 (116, 167) | -11 (-20, -1) | **0.047** |
| Fasting plasma glucose, mg/dL | 114 (108, 121) | 114 (108,121) | 114 (107, 121) | 109 (101, 115) | 109 (102, 116) | -2 (-5, 1) | 0.070 |
| HbA1C, % | 6.1 (5.8, 6.4) | 6.1 (5.9, 6.4) | 6.2 (5.9, 6.5) | 6.0 (5.7, 6.3) | 5.9 (5.6, 6.2) | -0.1 (-0.2, 0.0) | 0.152 |
| Number of MetS components (range: 0-5), # | 2.4 (2.1, 2.7) | 2.4 (2.1, 2.7) | 2.4 (2.1, 2.7) | 2.2 (1.8, 2.5) | 2.3 (2.0, 2.7) | -0.1 (-0.2, 0.1) | 0.382 |
| 24-hour urinary sodium, mg | 3662 (3312, 4009) | 3800 (3473, 4126) | 3395 (3002, 3742) | 3533 (3183, 3882) | 3636 (3259, 4014) | -48 (-184, 94) | 0.532 |
| 24-hour urinary potassium, mg | 1365 (1229, 1498) | 1505 (1381, 1634) | 1498 (1353, 1642) | 1431 (1295, 1568) | 1587 (1439, 1732) | 59 (4, 113) | 0.061 |
| Sodium screener score (range: 0-100) | 27.7 (25.9, 29.4) | 26.1 (24.4, 27.8) | 27.0 (25.1, 28.8) | 27.0 (25.1, 28.8) | 24.7 (22.8, 26.6) | -0.9 (-1.6, -0.2) | **0.040** |
| ***Binary outcomes (statistic: multivariable odds ratio, 95%CI)*** | | | | | | | |
| BMI ≥25 kg/m^2^ | REF | 1.15 (0.70, 1.90) | 1.55 (0.91, 2.66) | 1.10 (0.65, 1.87) | 1.00 (0.59, 1.70) | 0.98 (0.83, 1.16) | 0.950 |
| High MUAC | REF | 1.24 (0.75, 2.05) | 1.45 (0.85, 2.49) | 0.79 (0.46, 1.34) | 1.05 (0.62, 1.78) | 0.94 (0.79, 1.11) | 0.553 |
| Abdominal obesity | REF | 0.93 (0.56, 1.54) | 0.98 (0.57, 1.68) | 0.98 (0.57, 1.67) | 0.85 (0.50, 1.45) | 0.93 (0.79, 1.1) | 0.664 |
| Waist-to-height ratio >0.5 | REF | 0.83 (0.49, 1.41) | 1.23 (0.69, 2.19) | 0.80 (0.46, 1.40) | 0.72 (0.41, 1.25) | 0.88 (0.73, 1.05) | 0.272 |
| Hypertension | REF | 0.75 (0.45, 1.25) | 0.72 (0.41, 1.23) | 0.51 (0.29, 0.88) | 0.75 (0.44, 1.29) | 0.88 (0.74, 1.04) | 0.119 |
| Anemia | REF | 0.45 (0.22, 0.88) | 1.13 (0.60, 2.13) | 0.71 (0.37, 1.37) | 1.07 (0.57, 2.02) | 1.13 (0.91, 1.39) | 0.412 |
| Raised LDL-C | REF | 1.31 (0.77, 2.22) | 0.97 (0.55, 1.71) | 1.18 (0.68, 2.06) | 0.83 (0.47, 1.46) | 0.90 (0.76, 1.08) | 0.451 |
| Reduced HDL-C | REF | 0.97 (0.57, 1.64) | 0.78 (0.44, 1.38) | 0.51 (0.28, 0.92) | 0.98 (0.56, 1.71) | 0.90 (0.75, 1.08) | 0.291 |
| Raised triglyceride | REF | 1.08 (0.64, 1.81) | 1.16 (0.67, 2.02) | 1.02 (0.59, 1.76) | 1.14 (0.67, 1.96) | 1.01 (0.85, 1.20) | 0.732 |
| Raised FPG | REF | 1.20 (0.72, 2.02) | 1.56 (0.91, 2.71) | 1.14 (0.66, 1.96) | 1.00 (0.58, 1.74) | 1.03 (0.87, 1.23) | 0.939 |
| Raised HbA1C | REF | 0.80 (0.36, 1.77) | 1.23 (0.56, 2.74) | 0.78 (0.33, 1.82) | 0.80 (0.33, 1.87) | 0.94 (0.72, 1.23) | 0.631 |
| MetS | REF | 1.12 (0.66, 1.89) | 1.34 (0.77, 2.33) | 0.82 (0.47, 1.45) | 1.49 (0.87, 2.58) | 1.06 (0.89, 1.26) | 0.424 |

**Footnote:** Values presented as multivariable estimated marginal means or odds ratios (95% CI) associated with each metric quintile or per 1-SD positive difference in metrics. Models adjusted for age, sex, education, physical activity, smoking, sleep quality, and study group (Mahidol staff versus community sample). *p*-trend: multivariable p for linear trend across metric quintiles. Bold cells indicate *p*-trend <0.05. Abbreviations: GDQS, Global Diet Quality Score; 24HR / -24, 24-hour dietary recall; FFQ, food-frequency questionnaire; BMI: body-mass index; MUAC, mid-upper arm circumference; WC, waist circumference; SBP, systolic blood pressure; DPB, diastolic blood pressure; Hb, hemoglobin; LDL, low-density lipoprotein; HDL, high-density lipoprotein; TG, triglyceride; FPG, fasting plasma glucose; HbA1C, hemoglobin A1C; MetS, metabolic syndrome.

**Supplemental Table 7.** Multivariable associations between the GDQS-FFQ and outcomes related to nutrient adequacy and metabolic risk among Thai adults

| **Outcome** | **GDQS-FFQ quintile 1** | **GDQS-FFQ quintile 2** | **GDQS-FFQ quintile 3** | **GDQS-FFQ quintile 4** | **GDQS-FFQ quintile 5** | **Per 1 SD difference in GDQS-FFQ** | ***p*-trend** |
| --- | --- | --- | --- | --- | --- | --- | --- |
| ***Continuous outcomes (statistic: estimated marginal mean, 95%CI)*** | | | | | | | |
| Probability of protein adequacy computed from 24HR (energy-adjusted), % | 67.1 (60.9, 73.4) | 67.9 (61.3, 74.5) | 65.8 (59.1, 72.4) | 68.1 (61.1, 75.0) | 71.8 (64.8, 78.9) | 1.6 (-1.0, 4.2) | 0.308 |
| Mean probability of micronutrient adequacy computed from 24HR (energy-adjusted), % | 37.1 (34.9, 39.3) | 39.5 (37.2, 41.9) | 43.5 (36.6, 41.3) | 41.8 (39.4, 44.3) | 41.4 (38.9, 43.9) | 1.6 (0.7, 2.6) | **0.001** |
| Probability of protein adequacy computed from FFQ (energy-adjusted), % | 37.7 (31.3, 44.2) | 44.4 (37.6, 51.3) | 50.2 (43.3, 57.0) | 55.2 (48.0, 62.4) | 51.3 (44.1, 58.5) | 5.5 (2.8, 8.2) | **<0.001** |
| Mean probability of micronutrient adequacy computed from FFQ (energy-adjusted), % | 32.2 (28.9, 35.5) | 41.1 (37.7, 44.6) | 44.7 (41.2, 48.2) | 49.9 (46.2, 53.5) | 52.5 (48.9, 56.2) | 7.1 (5.8, 8.5) | **<0.001** |
| Body-mass index, kg/m^2^ | 25.6 (24.7, 26.6) | 25.9 (24.9, 26.9) | 25.8 (24.8, 26.8) | 25.7 (24.7, 26.7) | 25.7 (24.7, 26.7) | -3.4 (-42.0, 35.2) | 0.971 |
| Mid-upper arm circumference, cm | 30.8 (30.0, 31.6) | 31.0 (30.1, 31.8) | 31.0 (30.2, 31.9) | 30.6 (29.7, 31.5) | 30.9 (30.0, 31.7) | -0.1 (-0.4, 0.3) | 0.858 |
| Waist circumference, cm | 88.5 (86.2, 90.8) | 87.3 (84.9, 89.7) | 86.8 (84.4, 89.3) | 86.9 (84.3, 89.5) | 87.4 (84.8, 90.0) | -0.4 (-1.4, 0.5) | 0.442 |
| Fat mass, % | 31.5 (30.1, 32.8) | 30.8 (29.3, 32.2) | 30.6 (29.2, 32.0) | 30.0 (28.5, 31.6) | 29.9 (28.4, 31.4) | -0.6 (-1.1, 0.0) | 0.054 |
| Systolic blood pressure, mmHg | 125 (122, 128) | 125 (120, 127) | 125 (121, 128) | 126 (122, 129) | 124 (121, 128) | -1 (-2, 1) | 0.960 |
| Diastolic blood pressure, mmHg | 84 (81, 86) | 81 (79 (84) | 83 (81, 85) | 83 (80, 85) | 82.0 (79, 85) | -1 (-2, 0) | 0.580 |
| Hemoglobin, g/L | 134.5 (132.0, 137.1) | 134.5 (131.8, 137.0) | 136.7 (134.0, 139.4) | 136.9 (134.1, 139.8) | 135.0 (132.5, 138.2) | 0.3 (-0.8, 1.4) | 0.290 |
| Total cholesterol, mg/dL | 211 (203, 220) | 211 (202, 220) | 215 (206, 224) | 204 (194, 213) | 203 (194, 213) | -3 (-6, 18) | 0.076 |
| LDL-C, mg/dL | 145 (137, 153) | 148 (139, 156) | 148 (139, 157) | 141 (132, 150) | 138 (129, 147) | -2 (-5, 2) | 0.121 |
| HDL-C, mg/dL | 49 (47, 52) | 50 (47, 52) | 52 (50, 54) | 50 (47, 52) | 51 (49, 54) | 1 (-0, 2) | 0.234 |
| Triglyceride, mg/dL | 168 (145, 192) | 145 (120, 169) | 147 (123, 172) | 140 (114, 166) | 149 (123, 174) | -9 (-19, 0) | 0.204 |
| Fasting plasma glucose, mg/dL | 112 (105, 118) | 112 (105, 119) | 111 (104, 118) | 113 (106, 121) | 117 (109, 124) | 1 (-2, 4) | 0.264 |
| HbA1C, % | 6.1 (5.8, 6.3) | 6.1 (5.8, 6.4) | 6.1 (5.8, 6.4) | 6.0 (5.7, 6.3) | 6.2 (5.9, 6.5) | 0.0 (-0.1, 0.1) | 0.630 |
| Number of MetS components (range: 0-5), # | 2.5 (2.2, 2.8) | 2.2 (1.9, 2.5) | 2.3 (2.0, 2.6) | 2.4 (2.1, 2.7) | 2.2 (1.9, 2.6) | -0.1 (-0.2, 0.0) | 0.434 |
| 24-hour urinary sodium, mg | 3528 (3181, 3876) | 3800 (3445, 4154) | 3625 (3266, 3986) | 3489 (3135, 3843) | 3512 (3144, 3878) | -71 (-207, 74) | 0.503 |
| 24-hour urinary potassium, mg | 1287 (1154, 1420) | 1404 (1268, 1541) | 1564 (1427, 1700) | 1541 (1408, 1677) | 1603 (1463, 1743) | 98 (43, 156) | **<0.001** |
| Sodium screener score (range: 0-100) | 27.0 (25.3, 28.7) | 27.2 (25.4, 29.0) | 25.7 (23.9, 27.5) | 25.0 (23.1, 26.9) | 27.7 (25.7, 29.6) | -0.1 (-0.8, 0.6) | 0.723 |
| ***Binary outcomes (statistic: multivariable odds ratio, 95%CI)*** | | | | | | | |
| BMI ≥25 kg/m^2^ | REF | 0.85 (0.50, 1.44) | 0.83 (0.49, 1.40) | 0.79 (0.46, 1.34) | 0.69 (0.4, 1.18) | 0.87 (0.73, 1.03) | 0.180 |
| High MUAC | REF | 0.92 (0.54, 1.56) | 0.80 (0.47, 1.35) | 0.75 (0.44, 1.29) | 0.79 (0.46, 1.36) | 0.9 (0.75, 1.07) | 0.283 |
| Abdominal obesity | REF | 0.63 (0.36, 1.07) | 0.58 (0.34, 0.99) | 0.61 (0.35, 1.06) | 0.68 (0.39, 1.16) | 0.9 (0.75, 1.07) | 0.200 |
| Waist-to-height ratio >0.5 | REF | 0.66 (0.37, 1.15) | 0.59 (0.33, 1.02) | 0.63 (0.35, 1.11) | 0.81 (0.45, 1.44) | 0.94 (0.78, 1.12) | 0.479 |
| Hypertension | REF | 0.70 (0.40, 1.21) | 1.17 (0.68, 2.01) | 1.18 (0.68, 2.05) | 0.89 (0.51, 1.54) | 0.96 (0.8, 1.14) | 0.656 |
| Anemia | REF | 1.46 (0.77, 2.81) | 0.90 (0.45, 1.79) | 0.76 (0.37, 1.53) | 1.41 (0.74, 2.74) | 1.05 (0.85, 1.3) | 0.961 |
| Raised LDL-C | REF | 0.96 (0.55, 1.65) | 0.98 (0.56, 1.68) | 0.78 (0.44, 1.37) | 0.73 (0.42, 1.29) | 0.95 (0.79, 1.14) | 0.207 |
| Reduced HDL-C | REF | 0.77 (0.44, 1.35) | 0.59 (0.33, 1.04) | 1.19 (0.69, 2.07) | 0.85 (0.48, 1.49) | 0.96 (0.80, 1.16) | 0.881 |
| Raised triglyceride | REF | 1.05 (0.61, 1.79) | 1.05 (0.61, 1.79) | 0.77 (0.44, 1.33) | 0.81 (0.47, 1.40) | 0.88 (0.73, 1.05) | 0.243 |
| Raised FPG | REF | 0.62 (0.36, 1.06) | 0.99 (0.58, 1.68) | 0.89 (0.52, 1.54) | 0.71 (0.41, 1.23) | 0.95 (0.80, 1.14) | 0.621 |
| Raised HbA1C | REF | 0.87 (0.36, 2.00) | 0.97 (0.42, 2.22) | 1.03 (0.44, 2.39) | 1.36 (0.60, 3.06) | 1.08 (0.82, 1.42) | 0.407 |
| MetS | REF | 0.57 (0.33, 0.98) | 0.63 (0.36, 1.08) | 0.82 (0.47, 1.41) | 0.65 (0.37, 1.12) | 0.87 (0.72, 1.04) | 0.428 |

**Footnote:** Values presented as multivariable estimated marginal means or odds ratios (95% CI) associated with each metric quintile or per 1-SD positive difference in metrics. Models adjusted for age, sex, education, physical activity, smoking, sleep quality, and study group (Mahidol staff versus community sample). *p*-trend: multivariable p for linear trend across metric quintiles. Bold cells indicate *p*-trend <0.05. Abbreviations: GDQS, Global Diet Quality Score; MDDW, Minimum Dietary Diversity-Women; AHEI, Alternative Healthy Eating Index; GDR, Global Dietary Recommendations; 24HR, 24-hour dietary recall; FFQ / -FFQ, food-frequency questionnaire; BMI: body-mass index; MUAC, mid-upper arm circumference; WC, waist circumference; SBP, systolic blood pressure; DPB, diastolic blood pressure; Hb, hemoglobin; LDL, low-density lipoprotein; HDL, high-density lipoprotein; TG, triglyceride; FPG, fasting plasma glucose; HbA1C, hemoglobin A1C; MetS, metabolic syndrome.

**Supplemental Table 8:** Statistical comparison of multivariable associations between diet metrics and outcomes related to nutrient adequacy and metabolic risk among Thai adults: GDQS-24 vs. non-GDQS metrics scored using 24HR data

| **Outcome** | **GDQS-24** | | **MDDW-24** | | **AHEI-24** | | **GDR-24** | | ***p-*diff** | | |
| --- | --- | --- | --- | --- | --- | --- | --- | --- | --- | --- | --- |
|  | **Per 1 SD** | ***p*-trend** | **Per 1 SD** | ***p*-trend** | **Per 1 SD** | ***p*-trend** | **Per 1 SD** | ***p*-trend** | **GDQS-24 vs MDDW-24** | **GDQS-24 vs AHEI-24** | **GDQS-24 vs GDR-24** |
| ***Continuous outcomes (statistic: estimated marginal mean, 95%CI)*** | | | | | | | | | | | |
| Probability of protein adequacy computed from 24HR (energy-adjusted), % | 1.4 (-1.2, 4.0) | 0.405 | 1.5 (-1.0, 4.1) | 0.145 | 0.9 (-1.7, 3.5) | 0.352 | 0.0 (-2.5, 2.6) | 0.829 | 0.562 | 0.850 | 0.252 |
| Mean probability of micronutrient adequacy computed from 24HR (energy-adjusted), % | 2.3 (1.5, 3.2) | **<0.001** | 2.5 (1.6, 3.3) | **<0.001** | 1.8 (0.8, 2.7) | **0.001** | 2.1 (1.2, 3.0) | **<0.001** | 0.499 | 0.200 | 0.664 |
| Probability of protein adequacy computed from FFQ (energy-adjusted), % | 0.8 (-1.9, 3.6) | 0.769 | 2.7 (-0.1, 5.4) | 0.103 | 1.8 (-1.0, 4.6) | 0.539 | 1.5 (-1.2, 4.2) | 0.774 | 0.277 | 0.601 | 0.616 |
| Mean probability of micronutrient adequacy computed from FFQ (energy-adjusted), % | 1.9 (0.5, 3.4) | **0.009** | 2.6 (1.1, 4.1) | **0.002** | 3.1 (1.6, 4.6) | **0.002** | 3.3 (1.8, 4.7) | **<0.001** | 0.663 | 0.823 | 0.258 |
| Body-mass index, kg/m^2^ | -0.1 (-0.5, 0.2) | 0.788 | 0.1 (-0.3, 0.5) | 0.741 | -0.3 (-0.7, 0.1) | 0.369 | -0.1 (-0.5, 0.3) | 0.358 | 0.588 | 0.386 | 0.781 |
| Mid-upper arm circumference, cm | -0.2 (-0.5, 0.1) | 0.340 | -0.0 (-0.4, 0.3) | 0.317 | -0.2 (-0.5, 0.2) | 0.511 | -0.2 (-0.5, 0.2) | 0.105 | 0.588 | 0.881 | 0.721 |
| Waist circumference, cm | -0.6 (-1.5, 0.4) | 0.484 | 0.2 (-0.7, 1.2) | 0.678 | -0.8 (-1.8, 0.1) | 0.178 | -0.5 (-1.4, 0.5) | 0.199 | 0.486 | 0.382 | 0.745 |
| Fat mass, % | -0.4 (-1.0, 0.2) | 0.370 | -0.0 (-0.6, 0.5) | 0.425 | -0.8 (-1.4, -0.2) | **0.024** | -0.5 (-1.1, 0.0) | **0.028** | 0.537 | 0.241 | 0.313 |
| Systolic blood pressure, mmHg | -1 (-2, 1) | 0.686 | -0.5 (-1.9, 0.8) | 0.402 | -0.5 (-1.9, 0.9) | 0.857 | -0.4 (-1.7, 1.0) | 0.484 | 0.645 | 0.756 | 0.564 |
| Diastolic blood pressure, mmHg | -1 (-2, 0) | 0.127 | -0.5 (-1.38, 0.5) | 0.293 | -0.6 (-1.6, 0.3) | 0.310 | -0.7 (-1.6, 0.2) | 0.201 | 0.453 | 0.631 | 0.891 |
| Hemoglobin, g/L | -0.8 (-1.9, 0.2) | 0.367 | -0.1 (-0.2, 0.1) | 0.106 | 0.0 (-0.1, 0.1) | 0.634 | 0.0 (-0.1, 0.1) | 0.688 | 0.370 | 0.284 | 0.251 |
| Total cholesterol, mg/dL | -2 (-5, 2) | 0.339 | -4 (-8, -1) | **0.009** | -2 (-6, 1) | 0.223 | -4 (-8, -1) | **0.021** | 0.194 | 0.455 | 0.197 |
| LDL-C, mg/dL | -2 (-5, 2) | 0.469 | -4 (-7, -1) | **0.012** | -2 (-5, 1) | 0.222 | -3 (-7, -0) | 0.074 | 0.185 | 0.359 | 0.245 |
| HDL-C, mg/dL | 1 (-0, 2) | 0.280 | 1 (-0, 2) | 0.089 | 1 (0, 2) | **0.023** | 0 (-1, 1) | 0.990 | 0.371 | 0.271 | 0.446 |
| Triglyceride, mg/dL | -11 (-20, -1) | **0.047** | -4 (-13, 5) | 0.210 | -12 (-22, -3) | **0.020** | -6 (-16, 3) | 0.290 | 0.331 | 0.586 | 0.388 |
| Fasting plasma glucose, mg/dL | -2 (-5, 1) | 0.070 | -3 (-6, -0) | **0.042** | -2 (-5, 1) | 0.263 | -2 (-5, 1) | 0.301 | 0.630 | 0.643 | 0.522 |
| HbA1C, % | -0.1 (-0.2, 0.0) | 0.152 | - 0.1 (-0.2, 0.0) | 0.138 | -0.1 (-0.2, 0.0) | 0.281 | -0.1 (-0.2, 0.0) | 0.112 | 0.694 | 0.800 | 0.782 |
| Number of MetS components (range: 0-5), # | -0.1 (-0.2, 0.1) | 0.382 | -0.1 (-0.1, 0.1) | 0.195 | -0.1 (-0.2, 0.0) | 0.170 | -0.0 (-0.2, 0.1) | 0.606 | 0.475 | 0.296 | 0.703 |
| 24-hour urinary sodium, mg | -48 (-184, 94) | 0.532 | 58 (-69, 193) | 0.569 | -30 (-161, 113) | 0.879 | -101 (-230, 35) | 0.311 | 0.305 | 0.781 | 0.460 |
| 24-hour urinary potassium, mg | 59 (4, 113) | 0.061 | 47 (0, 101) | 0.162 | 144 (90, 199) | **<0.001** | 86 (31, 137) | **0.003** | 0.558 | 0.179 | 0.425 |
| Sodium screener score (range: 0-100) | -0.9 (-1.6, -0.2) | **0.040** | -0.5 (-1.2, 0.2) | 0.292 | -3.8 (-4.4, -3.1) | **<0.001** | -2.1 (-2.8, -1.4) | **<0.001** | 0.213 | **0.008** | 0.060 |
| ***Binary outcomes (statistic: multivariable odds ratio, 95%CI)*** | | | | | | | | | | | |
| BMI ≥25 kg/m^2^ | 0.98 (0.83, 1.16) | 0.950 | 1.04 (0.88, 1.22) | 0.514 | 0.89 (0.75, 1.06) | 0.265 | 0.97 (0.82, 1.14) | 0.562 | 0.540 | 0.316 | 0.838 |
| High MUAC | 0.94 (0.79, 1.11) | 0.553 | 0.9 (0.76, 1.07) | **0.023** | 0.94 (0.79, 1.12) | 0.902 | 0.92 (0.78, 1.09) | 0.267 | 0.189 | 0.529 | 0.516 |
| Abdominal obesity | 0.93 (0.79, 1.1) | 0.664 | 1 .00 (0.85, 1.19) | 0.303 | 0.86 (0.72, 1.03) | 0.064 | 0.92 (0.78, 1.09) | 0.166 | 0.636 | 0.236 | 0.659 |
| Waist-to-height ratio >0.5 | 0.88 (0.73, 1.05) | 0.272 | 0.94 (0.79, 1.11) | 0.096 | 0.81 (0.68, 0.97) | **0.009** | 0.88 (0.74, 1.05) | **0.034** | 0.739 | 0.251 | 0.560 |
| Hypertension | 0.88 (0.74, 1.04) | 0.119 | 0.92 (0.78, 1.1) | 0.28 | 0.98 (0.82, 1.17) | 0.827 | 0.95 (0.8, 1.12) | 0.326 | 0.600 | 0.448 | 0.805 |
| Anemia | 1.13 (0.91, 1.39) | 0.412 | 0.97 (0.78, 1.2) | 0.881 | 0.99 (0.8, 1.22) | 0.977 | 0.95 (0.78, 1.17) | 0.584 | 0.725 | 0.434 | 0.306 |
| Raised LDL-C | 0.90 (0.76, 1.08) | 0.451 | 0.85 (0.71, 1.02) | 0.118 | 1.00 (0.84, 1.20) | 0.559 | 0.88 (0.74, 1.05) | 0.139 | 0.365 | 0.800 | 0.338 |
| Reduced HDL-C | 0.90 (0.75, 1.08) | 0.291 | 0.97 (0.81, 1.16) | 0.641 | 0.91 (0.76, 1.10) | 0.234 | 1.06 (0.89, 1.27) | 0.325 | 0.885 | 0.368 | 0.234 |
| Raised triglyceride | 1.01 (0.85, 1.20) | 0.732 | 0.97 (0.82, 1.15) | 0.670 | 0.96 (0.80, 1.14) | 0.791 | 0.98 (0.83, 1.17) | 0.528 | 0.397 | 0.487 | 0.616 |
| Raised FPG | 1.03 (0.87, 1.23) | 0.939 | 0.91 (0.77, 1.08) | 0.389 | 0.87 (0.73, 1.04) | 0.246 | 0.94 (0.79, 1.11) | 0.462 | 0.390 | 0.289 | 0.487 |
| Raised HbA1C | 0.94 (0.72, 1.23) | 0.631 | 0.86 (0.65, 1.13) | 0.444 | 0.98 (0.73, 1.29) | 0.939 | 0.92 (0.70, 1.20) | 0.414 | 0.639 | 0.578 | 0.710 |
| MetS | 1.06 (0.89, 1.26) | 0.424 | 1.03 (0.86, 1.22) | 0.737 | 0.92 (0.77, 1.11) | 0.466 | 1.02 (0.86, 1.22) | 0.546 | 0.248 | 0.139 | 0.484 |

**Footnote:** Values presented as multivariable estimated marginal means or odds ratios (95% CI) associated with a 1-SD positive difference in metrics. Models adjusted for age, sex, education, physical activity, smoking, sleep quality, and study group (Mahidol staff versus community sample). *p*-trend: multivariable p for linear trend across metric quintiles. *p*-diff: *p* for difference in linear trends across metric quintiles from Wald test. Bold cells indicate *p*-trend or *p*-diff <0.05. Abbreviations: GDQS, Global Diet Quality Score; 24HR / -24, 24-hour dietary recall; FFQ, food-frequency questionnaire; BMI: body-mass index; MUAC, mid-upper arm circumference; WC, waist circumference; SBP, systolic blood pressure; DPB, diastolic blood pressure; Hb, hemoglobin; LDL, low-density lipoprotein; HDL, high-density lipoprotein; TG, triglyceride; FPG, fasting plasma glucose; HbA1C, hemoglobin A1C; MetS, metabolic syndrome.

**Supplemental Table 9:** Statistical comparison of multivariable associations between diet metrics and outcomes related to nutrient adequacy and metabolic risk among Thai adults: GDQS-FFQ vs. non-GDQS metrics scored using FFQ data

| **Outcome** | **GDQS-FFQ** | | **MDDW-FFQ** | | | **AHEI-FFQ** | | | ***p*-diff** | |
| --- | --- | --- | --- | --- | --- | --- | --- | --- | --- | --- |
|  | **Per 1 SD** | ***p*-trend** | **Per 1 SD** | ***p*-trend** | **Per 1 SD** | | ***p*-trend** | ***GDQS-FFQ vs MDDW-FFQ*** | | ***GDQS-FFQ vs AHEI-FFQ*** |
| ***Continuous outcomes (statistic: estimated marginal mean, 95%CI)*** | | | | | | | | | | |
| Probability of protein adequacy computed from 24HR (energy-adjusted), % | 1.6 (-1.0, 4.2) | 0.308 | 1.8 (-0.8, 4.4) | 0.464 | 1.5 (-1.2, 4.2) | | 0.407 | 0.515 | | 0.578 |
| Mean probability of micronutrient adequacy computed from 24HR (energy-adjusted), % | 1.6 (0.7, 2.6) | **0.001** | 1.2 (0.3, 2.1) | 0.251 | 1.4 (0.5, 2.4) | | **<0.001** | 0.143 | | 0.798 |
| Probability of protein adequacy computed from FFQ (energy-adjusted), % | 5.5 (2.8, 8.2) | **<0.001** | 4.6 (1.9, 7.3) | **0.029** | 4.0 (1.2, 6.8) | | **0.013** | 0.330 | | 0.159 |
| Mean probability of micronutrient adequacy computed from FFQ (energy-adjusted), % | 7.1 (5.8, 8.5) | **<0.001** | 6.0 (4.6, 7.4) | **<0.001** | 6.2 (4.8, 7.7) | | **<0.001** | 0.112 | | 0.064 |
| Body-mass index, kg/m^2^ | -3.4 (-42.0, 35.2) | 0.971 | -0.2 (-0.6, 0.2) | 0.940 | -0.3 (-0.7, 0.1) | | 0.074 | 0.501 | | 0.139 |
| Mid-upper arm circumference, cm | -0.1 (-0.4, 0.3) | 0.858 | -0.2 (-0.5, 0.2) | 0.952 | -0.2 (-0.5, 0.1) | | 0.179 | 0.506 | | 0.247 |
| Waist circumference, cm | -0.4 (-1.4, 0.5) | 0.442 | -0.7 (-1.7, 0.2) | 0.722 | -1.2 (-2.2, -0.2) | | **0.011** | 0.917 | | 0.138 |
| Fat mass, % | -0.6 (-1.1, 0.0) | 0.054 | -0.6 (-1.2, -0.0) | 0.332 | -1.1 (-1.6, -0.5) | | **<0.001** | 0.354 | | 0.135 |
| Systolic blood pressure, mmHg | -1 (-2, 1) | 0.960 | -0.9 (-2.2, 0.5) | 0.136 | -0.9 (-2.3, 0.5) | | 0.181 | 0.197 | | 0.199 |
| Diastolic blood pressure, mmHg | -1 (-2, 0) | 0.580 | -0.9 (-1.9, -0.0) | 0.060 | -1.0 (-2.0, -0.0) | | 0.086 | 0.205 | | 0.262 |
| Hemoglobin, g/L | 0.3 (-0.8, 1.4) | 0.290 | 0.0 (-0.1, 0.1) | 0.863 | 0.1 (-0.0, 0.2) | | 0.100 | 0.422 | | 0.377 |
| Total cholesterol, mg/dL | -3 (-6, 1) | 0.076 | -3 (-6, 1) | 0.400 | -3 (-7, 0) | | 0.208 | 0.286 | | 0.476 |
| LDL-C, mg/dL | -2 (-5, 2) | 0.121 | -1 (-5, 2) | 0.739 | -2 (-6, 1) | | 0.278 | 0.201 | | 0.480 |
| HDL-C, mg/dL | 1 (-0, 2) | 0.234 | 1 (-0, 2) | 0.274 | 1 (0, 2) | | **0.032** | 0.742 | | 0.320 |
| Triglyceride, mg/dL | -9 (-19, 0) | 0.204 | -14 (-24, -5) | 0.078 | -14 (-24, -4) | | **0.012** | 0.304 | | 0.160 |
| Fasting plasma glucose, mg/dL | 1 (-2, 4) | 0.264 | 2 (-1, 5) | 0.072 | -1 (-4, 2) | | 0.272 | 0.397 | | 0.084 |
| HbA1C, % | 0.0 (-0.1, 0.1) | 0.630 | 0.1 (-0.1, 0.2) | 0.227 | -0.1 (-0.2, 0.0) | | 0.081 | 0.412 | | 0.083 |
| Number of MetS components (range: 0-5), # | -0.1 (-0.2, 0.0) | 0.434 | -0.1 (-0.3, -0.0) | 0.129 | -0.1 (-0.3, -0.0) | | **0.012** | 0.390 | | 0.166 |
| 24-hour urinary sodium, mg | -71 (-207, 74) | 0.503 | -71 (-207, 71) | 0.289 | -106 (-253, 48) | | 0.058 | 0.472 | | 0.181 |
| 24-hour urinary potassium, mg | 98 (43, 156) | **<0.001** | 70 (12, 125) | 0.093 | 156 (101, 215) | | **<0.001** | 0.129 | | 0.193 |
| Sodium screener score (range: 0-100) | -0.1 (-0.8, 0.6) | 0.723 | 0.5 (-0.2, 1.2) | **0.004** | -3.4 (-4.1, -2.7) | | **<0.001** | 0.083 | | **<0.001** |
| ***Binary outcomes (statistic: multivariable odds ratio, 95%CI)*** | | | | | | | | | | |
| BMI ≥25 kg/m^2^ | 1.31 (0.87, 1.98) | 0.180 | 0.78 (0.65, 0.92) | 0.102 | 0.81 (0.68, 0.97) | | **0.010** | 0.414 | | 0.237 |
| High MUAC | 1.12 (0.73, 1.73) | 0.283 | 0.9 (0.76, 1.07) | 0.56 | 0.88 (0.73, 1.05) | | 0.083 | 0.655 | | 0.407 |
| Abdominal obesity | 1.09 (0.71, 1.69) | 0.200 | 0.8 (0.67, 0.96) | 0.246 | 0.8 (0.67, 0.96) | | **0.004** | 0.612 | | 0.205 |
| Waist-to-height ratio >0.5 | 0.94 (0.78, 1.12) | 0.479 | 0.84 (0.70, 1.01) | 0.419 | 0.78 (0.65, 0.94) | | **0.007** | 0.359 | | 0.136 |
| Hypertension | 1.03 (0.66, 1.63) | 0.656 | 0.85 (0.71, 1.01) | 0.085 | 0.96 (0.8, 1.15) | | 0.575 | 0.134 | | 0.329 |
| Anemia | 0.91 (0.57, 1.44) | 0.961 | 1.02 (0.82, 1.27) | 0.447 | 1 (0.8, 1.25) | | 0.740 | 0.711 | | 0.321 |
| Raised LDL-C | 0.95 (0.79, 1.14) | 0.207 | 0.95 (0.79, 1.13) | 0.963 | 0.88 (0.73, 1.06) | | 0.223 | 0.219 | | 0.796 |
| Reduced HDL-C | 0.96 (0.80, 1.16) | 0.881 | 0.98 (0.82, 1.17) | 0.920 | 0.89 (0.74, 1.08) | | 0.218 | 0.636 | | 0.209 |
| Raised triglyceride | 0.88 (0.73, 1.05) | 0.243 | 0.88 (0.74, 1.05) | 0.219 | 0.82 (0.68, 0.99) | | **0.019** | 0.600 | | 0.176 |
| Raised FPG | 0.95 (0.80, 1.14) | 0.621 | 0.96 (0.81, 1.15) | 0.553 | 0.94 (0.78, 1.12) | | 0.583 | 0.571 | | 0.658 |
| Raised HbA1C | 1.08 (0.82, 1.42) | 0.407 | 1.06 (0.81, 1.39) | 0.352 | 0.95 (0.71, 1.26) | | 0.862 | 0.941 | | 0.206 |
| MetS | 0.87 (0.73, 1.03) | 0.428 | 0.8 (0.67, 0.95) | 0.061 | 0.8 (0.66, 0.96) | | **0.013** | 0.264 | | 0.173 |

**Footnote:** Values presented as multivariable estimated marginal means or odds ratios (95% CI) associated with a 1-SD positive difference in metrics. Models adjusted for age, sex, education, physical activity, smoking, sleep quality, and study group (Mahidol staff versus community sample). *p*-trend: multivariable p for linear trend across metric quintiles. *p*-diff: *p* for difference in linear trends across metric quintiles from Wald test. Bold cells indicate *p*-trend or *p*-diff <0.05. Abbreviations: GDQS, Global Diet Quality Score; MDDW, Minimum Dietary Diversity-Women; AHEI, Alternative Healthy Eating Index; GDR, Global Dietary Recommendations; 24HR, 24-hour dietary recall; FFQ / -FFQ, food-frequency questionnaire; BMI: body-mass index; MUAC, mid-upper arm circumference; WC, waist circumference; SBP, systolic blood pressure; DPB, diastolic blood pressure; Hb, hemoglobin; LDL, low-density lipoprotein; HDL, high-density lipoprotein; TG, triglyceride; FPG, fasting plasma glucose; HbA1C, hemoglobin A1C; MetS, metabolic syndrome.

**Supplemental Figure 1:** Participant flow chart and sequence of assessments.


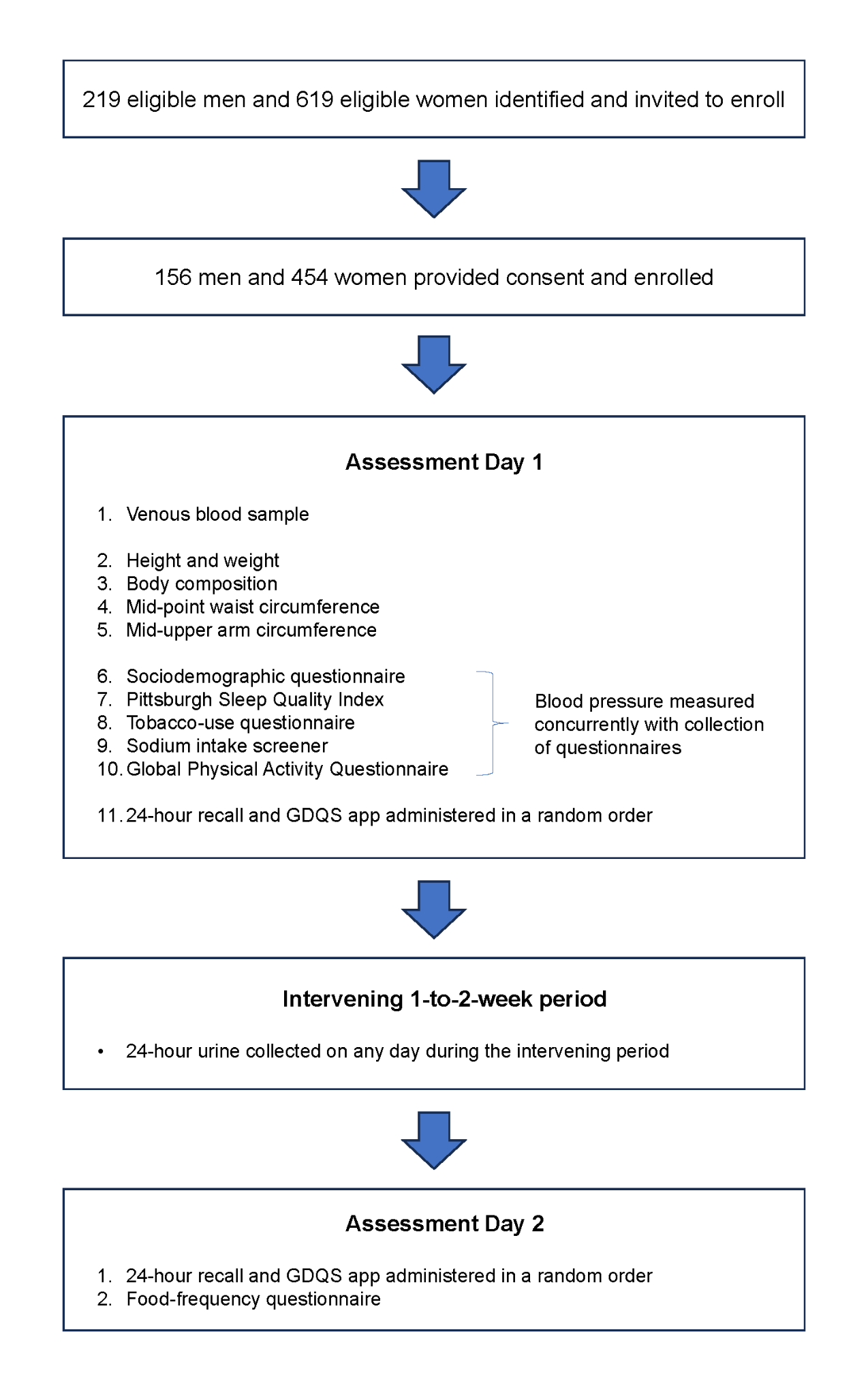

Supplement: Multimedia component 1 [file mmc1.docx]
